# Novel murine model of human astrovirus infection reveals cardiovascular tropism** **

**DOI:** 10.1128/jvi.00240-25

**Published:** 2025-04-30

**Authors:** Macee C. Owen, Yuefang Zhou, Holly Dudley, Taylor Feehley, Ashley Hahn, Christine C. Yokoyama, Margaret L. Axelrod, Chieh-Yu Lin, David Wang, Andrew B. Janowski

**Affiliations:** 1 Immunology Program, Washington University School of Medicine 12275 , St. Louis, Missouri, USA; 2 Department of Pediatrics, Division of Pediatric Infectious Diseases, Washington University School of Medicine 12275 , St. Louis, Missouri, USA; 3 Vaccine and Infectious Disease Division, Fred Hutchinson Cancer Center, Seattle, Washington, USA; 4 Xanadu Bio, Gladwyne, Pennsylvania, USA; 5 Department of Internal Medicine, Division of Dermatology, Washington University School of Medicine 12275 , St. Louis, Missouri, USA; 6 Department of Pathology and Immunology, Washington University School of Medicine, St. Louis, Missouri, USA; 7 Department of Molecular Microbiology, Washington University School of Medicine 12275 , St. Louis, Missouri, USA; Emory University School of Medicine, Atlanta, Georgia, USA

**Keywords:** virology, astrovirus, animal models, myocarditis, tropism, viral immunity

## Abstract

Astroviruses are a common cause of gastrointestinal disease in humans and have been linked to fatal cases of encephalitis. A major barrier to the study of human-infecting astroviruses is the lack of an *in vivo* model as previous attempts failed to identify a host that supports viral replication. We describe a novel murine model of infection using astrovirus VA1/HMO-C (VA1), an astrovirus with high seroprevalence in humans. VA1 is cardiotropic, and viral RNA levels peak in the heart tissue 7 days post-inoculation in multiple different murine genetic backgrounds. Infectious VA1 particles could be recovered from heart tissue 3 and 5 days post-inoculation. Viral capsid was detected intracellularly in the heart tissue by immunostaining, and viral RNA was detected in cardiac myocytes, endocardium, and endothelial cells based on fluorescent *in situ* hybridization and confocal microscopy. Histologically, we identified inflammatory infiltrates consistent with myocarditis in some mice, with viral RNA colocalizing with the infiltrates. These foci contained CD3 +T cells and CD68 +macrophages. Viral RNA levels increased by >10 fold in the heart tissue or serum samples from Rag1 or Stat1 knockout mice, demonstrating the role of both adaptive and innate immunity in the response to VA1 infection. Based on the *in vivo* tropisms, we tested cardiac-derived primary cells and determined that VA1 can replicate in primary human cardiac endothelial cells, suggesting a novel cardiovascular tropism in human cells. This novel *in vivo* model of a human-infecting astrovirus enables further characterization of the host immune response and reveals a new cardiovascular tropism of astroviruses.

**IMPORTANCE:**

Astroviruses routinely cause infections in humans; however, few methods were available to study these viruses. Here, we describe the first animal system to study human-infecting astroviruses by using mice. We demonstrate that mice are susceptible to astrovirus VA1, a strain that commonly infects humans and has been linked to fatal brain infections. The virus infects the heart tissue and is associated with inflammation. When mice with impaired immune systems were infected with VA1, they were found to have higher amounts of the virus in their hearts and blood. We found that VA1 can infect cells from human blood vessels of the heart, which is associated with human health. This model will enable us to better understand how astroviruses cause disease and how the immune system responds to infection. Our findings also suggest that astroviruses could be linked to cardiovascular diseases, including in humans.

## INTRODUCTION

Astroviruses are a diverse family of RNA viruses that are frequently detected from many vertebrate species, including mammals, birds, reptiles, amphibians, and fish (1). Initially discovered in 1975 from an outbreak of gastroenteritis, astroviruses have been primarily detected from stool specimens and wastewater samples (2, 3). The causal link between their presence and causing gastrointestinal (GI) disease was confirmed by oral challenge studies in humans and other mammals (4–8). Further epidemiological studies have established astroviruses as a common etiological agent of gastroenteritis, with some estimates nearing 6 million new human cases each year (9).

Since 2010, astroviruses have received additional attention due to the recognition of their capacity to cause central nervous system (CNS) diseases. In humans, 17 cases with a mortality rate of approximately 50% have been reported (1, 10–13). Similar discoveries have been reported in other mammals infected by other astrovirus genotypes, including in cattle, pigs, minks, alpaca, musk ox, and sheep (14–20). Currently, astroviruses are not part of routine clinical testing, so the disease burden induced by astroviruses in neurological diseases in humans and other mammals is poorly understood.

In addition to the newly described neurotropism, there is increased recognition that astroviruses have additional tissue tropisms (1). Avian astroviruses have been linked to fatal liver and kidney diseases that are of significance to the poultry industry (21–23). In humans, astroviruses have been implicated as a possible cause of hepatitis and inducing an increased risk of immune thrombocytopenia (24, 25). Astroviruses have also been detected from the respiratory tract; however, further data are needed to clarify whether there is a causal relationship between astrovirus infection and respiratory diseases (15, 26–35). Other potential tissue tropisms have been described, including detection of viral nucleic acids from the heart tissue in ducks, chickens, geese, pigs, and humans (36–40). However, it is unclear as to what extent these findings are incidental findings versus if they reflect *bona fide* cardiovascular infections.

Three clades of astroviruses are recognized to cause disease in humans; the classic human astrovirus (HAstV), MLB, and VA clades (3). Most humans have been exposed to these clades and develop neutralizing antibodies as >50% of adults have a serological response to at least one species within each clade (41–47). All three clades are frequently detected from human stool samples from cases of diarrhea or gastroenteritis (3). In addition, they all have been associated with meningoencephalitis in humans, with astrovirus VA1 (VA1) being the most frequently detected strain (10–13, 26, 30, 39, 48–54).

Despite the significant role of astroviruses in human health, there are only limited experimental systems to study the pathogenesis of disease, including the lack of any *in vivo* model of infection for a human-infecting astrovirus. One group described their attempts to use human-infecting astrovirus strains to inoculate mice, but there was no clear evidence of viral replication or virus-induced disease in the mice (55). Other models using nonhuman-infecting astroviruses have provided important insights into viral pathogenicity. A turkey astrovirus strain causes diarrhea in a turkey poultry model, with viral RNA detected in many tissues including the liver, spleen, kidneys, bone marrow, and plasma (56, 57). Replication was hypothesized to be restricted to the GI tract based on the detection of intracellular RNA from *in situ* hybridization assays (56, 57). Murine astrovirus strains have also been detected from mice and used as an experimental model system (55, 58–61). However, the murine astrovirus model has limitations. Most laboratory and commercially available mice are infected with murine astrovirus, and viral infection does not cause any apparent disease (55, 58, 60).

Given the limitations of current animal models, we sought to develop a murine model of infection using a human-infecting astrovirus. We used a previously propagated VA1 genotype for inoculation into mice (62, 63). Unexpectedly, we identified viral RNA and viral capsid and isolated infectious viral particles from the heart tissue that was associated with inflammatory infiltrates. This novel model of human astrovirus infection overcomes a major limitation in the study of astroviruses and enables further study of the pathogenesis of infection. Furthermore, it demonstrates a previously uncharacterized cardiovascular tropism for astroviruses.

## MATERIALS AND METHODS

### Animals

A/J (strain #000646), C57BL/6 (strain #000664), C3H/HeJ (strain #000659), Balb/c (strain #000651), J:ARC(S) (strain #034608), B6.129S7-Rag1^tm1Mom^/J (strain #002216), and B6.129S(Cg)-Stat1^tm1Dlv^/J (strain #012606) mice were obtained from the Jackson Laboratory. These mice were routinely screened by the vendor for murine norovirus infection by serology testing, but no screening is performed for murine astrovirus. All mice were maintained in a specific-pathogen-free facility (BSL2) following institutional guidelines and with protocols approved by the AAALAC-accredited Animal Studies Committee at Washington University in St Louis. All animals were maintained on 12 hour light cycles and housed at 21°C and 50% humidity. Experiments were performed with mice at 5–6 weeks of age and were carried out utilizing BSL2 conditions. Mice were given *ad libitum* access to food and water.

### Cell culture

All cell lines and infection steps were carried out at 37°C with 5% CO2. The undifferentiated human intestinal epithelial cell line (Caco-2) was cultured in the growth medium consisting of Dulbecco’s modified Eagle medium (DMEM) with L-glutamine supplemented with 10% fetal bovine serum (FBS; Gibco) and 1% of 10,000 units/mL of penicillin and streptomycin (Gibco). Primary cell lines were purchased from Sciencell, including primary human cardiac microvascular endothelial cells (CMECs), human coronary artery endothelial cells (HCAECs), human umbilical vein endothelial cells (HUVECs), human hepatic sinusoidal endothelial cells (HHSECs), human cardiac myocytes, and C57BL/6 murine cardiac myocytes. C57BL/6 mouse primary cardiac microvascular endothelial cells were purchased from Cell Biologics. All primary cells were cultured using the media provided by the vendor, including the cardiac myocyte medium-serum free for cardiac myocytes (Sciencell) and endothelial cell medium (Sciencell and Cell Biologics). Infection of cells was performed as described previously using a multiplicity of infection of 3 for all other cell lines (63). Cell fractions were collected at reported time points post-inoculation in TRIzol for RNA extraction.

### Viral stock

We used a 0.2 µm sterile-filtered VA1 viral stock that was passaged in Caco-2 cells (C-P8). The stock was generated using the same methods as previously described (63). The stock did not have any mutations relative to a previously described stock (C-P7). The stock has 13.29 RNA copies for every one infectious virus particle.

### Murine time course experiment of VA1 inoculation

A/J mice were inoculated intraperitoneally (IP) with 1.5 × 10^7^ focus-forming units (FFU) of C-P8 in 200 µL of DMEM. Mock-infected mice were IP inoculated with 200 µL of the cell lysate in DMEM containing no virus. At least three independent infections of mice were performed, typically in groups of three to five mice per experiment. Mice of both sexes in similar ratios were tested for all experiments. Mice were monitored daily for changes in weight and clinical signs of infection. Mice were euthanized on days 3, 5, 7, 14, or 21 post-inoculation (p.i.), and tissues were harvested.

### VA1 inoculation in mice of different genetic backgrounds and routes

C57BL/6, Balb/c, C3H/HeJ, J:ARC(S) (Swiss-outbred), B6.129S7-Rag1^tm1Mom^/J, and B6.129S(Cg)-Stat1^tm1Dlv^/J were inoculated IP with 1.5 × 10^7^ FFU of C-P8 in 200 µL of DMEM, as previously performed for A/J mice. Inoculations were performed in three independent experiments of three mice per experiment. Mice were euthanized, and tissues were harvested at day 7 p.i. as described in the methods below.

Mice were also infected by different routes. For the *per os* route (PO), 100 µL was slowly pipetted into the mouths of the mice. The mice were allowed to lick and swallow the liquid until the full volume was delivered. For oral gastric lavage (OG), a lavage needle was inserted into the oropharynx of the mice and passed until it entered the stomach. A volume of 200 µL was then administered. For intracranial inoculations (IC), mice were anesthetized with a ketamine/xylazine cocktail (100 mg/kg for ketamine; 10 mg/kg for xylazine). Once the mice were sufficiently sedated, 20 µL (1.5 × 10^6^ FFU) of the inoculum was delivered to the mouse central nervous system by insertion of an insulin needle into the cranium. After insertion, mice were monitored for bleeding, neurological deficits, and recovery from the anesthesia cocktail.

### Tissue collection

Mice were euthanized with CO2 and subsequent cervical dislocation. Blood was obtained from the inferior vena cava via venipuncture. Apexes of hearts were collected for RNA isolation (~30 mg), and the remainder of the heart was transversely sectioned and formalin-fixed for subsequent histopathological analysis. A small section of the left lobe of the liver was excised for RNA isolation, and the entire median lobe of the liver was removed and formalin-fixed for histopathological analysis. In addition to the heart and liver, small tissue sections were collected from the following organs: brain, brainstem, lung, kidney, spleen, ileum, colon, mesenteric lymph node, and skeletal muscle. A stool sample was also obtained from the intestinal tract. All tissue samples were weighed and stored at −80°C. Blood was allowed to clot for 30 minutes prior to centrifugation at 6,000 RPM for 3 minutes in blood collection tubes (BD), and the serum was aliquoted into separate tubes for storage at −80°C.

### RNA isolation

Tissue samples were bead-homogenized using a BeadBlaster (BioSpec) in 1 mL of phosphate-buffered saline (PBS) and then centrifuged. RNA was isolated from 100 µL of the sample supernatant using TRIzol (ThermoFisher) in the Direct-zol 96 kit (Zymo Research) and stored at −80°C.

### qRT-PCR

A previously published (62) quantitative reverse transcription-PCR (qRT-PCR) was performed to quantify viral RNA from tissue. Isolated RNA was combined with primers, probe, and TaqMan Fast Virus 1-Step master mix (Applied Biosystems) and analyzed on a ViiA 7 or QuantStudio 3 Real-Time PCR systems (Applied Biosystems). Viral copy numbers were calculated and normalized to the tissue weight by dividing the copy number by milligrams of organ weight. For all tissues, the detected copy number was normalized to tissue weight.

### Focus-forming assay

Focus forming assays (FFAs) were performed to quantify VA1 titers as previously described (42). Homogenized tissue suspended in PBS was serially diluted for inoculation into Caco-2 cells. The cells were then fixed and stained with the infectious titer quantified.

### Histology

Hearts and livers were collected and fixed in 10% neutral-buffered formalin for 48–72 hours. The tissues were then placed in histology cassettes and dehydrated through a graded series of 70%, 90%, and 100% ethyl alcohol and then two rounds of xylene washes. Tissues were then paraffin-embedded and sectioned at 4 µM thickness. Tissues were stained with hematoxylin and eosin, examined for any pathological abnormality via light microscopy at 20X magnification, and images were obtained using a Zeiss Cell Observer inverted microscope. Images were edited using the Zen 2.3 lite application (Carl Zeiss Microscopy). Tissue selection procedures were performed under blinded conditions and then independently reviewed for the presence of foci of inflammatory cells by two pathologists. The scoring system for the foci was as follows: 0 = no foci present in the section, 1 = one or two foci present in the section, 2 = three or more inflammatory foci present in the section.

### Immunofluorescent microscopy

Seven days post-inoculation, mice were anesthetized with ketamine (100 mg/kg) and xylazine (10 mg/kg) before being perfused first with PBS to rinse out the blood and then fixed with 4% paraformaldehyde (PFA) in PBS (Sigma). The hearts were dissected and post-fixed by immersing in 4% PFA for an additional 3 hours before being cryo-protected by incubation in 15% and 30% sucrose in PBS. The samples were then embedded in Tissue-Tek OCT compound (Electron Microscopy Sciences), and longitudinal sections were cut using a cryostat (Cryotome; Leica). Sections were initially washed with PBS, permeabilized with 0.1% Triton-X in PBS, blocked with 5% goat serum in PBS for 1 hour at room temperature, and then incubated with primary antibodies to mouse anti-VA1 capsid (Mab2A2; 1:2000) (42, 64), rat anti-CD45 (1:20, BD Pharmingen, #550539), FITC rat anti-CD3 (1:50, Invitrogen, #11–0032-82), or Alexa Fluor 488 rat anti-CD68 (1:200; Biolegend #137011) overnight at 4°C. For anti-VA1 capsid staining, the tissue was first blocked using Mouse on Mouse immunodetection kit (Vector Laboratories), using two drops in 1.5 mL of blocking buffer. The sections were incubated with Alexa Flour 488 goat anti-mouse secondary antibody (Invitrogen) at a dilution of 1:1000 for 1 hour at room temperature. For CD45 staining, sections were further incubated with Alexa Fluor 488 goat anti-rat secondary antibody (Invitrogen) at a dilution of 1:1000 for 1 hour at room temperature. Sections were counterstained with 4,6-diamidino-2-phenylindole (DAPI, Sigma) and imaged at 20–40 x using a Zeiss Cell Observer inverted microscope. Images were edited using the Zen 2.3 lite application (Carl Zeiss Microscopy). Four mice from each group were analyzed. A total of five representative images of each heart were obtained, and fluorescent cells were counted using ImageJ (65). The number of positive cells were normalized to per 1,000 of DAPI-positive nuclei.

### Fluorescent *in situ* hybridization (FISH)

FISH was performed on murine formalin-fixed-paraffin-embedded tissues using RNAscope Multiplex Fluorescent Reagent Kit v2 assay (Advanced Cell Diagnostics; ACDBio) based on the manufacturer’s instructions. As a positive control, VA1-infected or mock-infected Caco-2 cells were collected and suspended in 1% melted agarose. The block was allowed to cool and solidify prior to embedding in paraffin. After sectioning, tissue sections were deparaffinized in xylene and then dehydrated in 100% ethyl alcohol. Tissues were pretreated with 3% hydrogen peroxide, and antigen retrieval was performed in a pressure cooker at 100°C for 15 minutes using RNAscope Target Retrieval Agent diluted to 1X in deionized water. Tissues were treated with RNAscope Protease Plus at 40°C for 30 minutes in an ACDBio RNAscope oven, using the recommended humidity control tray. Targeted RNA sequences were then hybridized using the following target probes obtained from ACDBio: RNAscope Probe V-Astrovirus-VA1-ORF2-C1 (854601-C1; positive-sense detecting region of ORF2; reference genome accession number NC_013060.1), RNAscope Probe-Mm-Vwf-C3 (858851-C3), and RNAscope Probe-Mm-Ryr2-C2 (479981-C2). The VA1 probe was designed to not cross-react with other astroviruses, including murine astrovirus. Positive (RNAscope 3-plex Positive Control Probe-Mm) and negative (RNAscope 3-plex Negative Control Probe-Mm) control probes were utilized to validate the assay. All probes were diluted as per the manufacturer’s instructions in Probe Diluent (ACDBio). After washing with 1 x RNAscope Wash Buffer in deionized water, amplification steps were then performed using the RNAscope Multiplex FL v2 AMP1, -2, and -3 as per the manufacturer’s instructions. Fluorophores obtained from Akoya Biosciences (Opal 520: FP1487001KT, Opal 570: FP1488001KT, Opal 650: FP1497001KT) were reconstituted in DMSO and were diluted at 1:1500 in RNAscope TSA buffer on the same day as intended for use. After amplification steps, Opal 520, 570, and 650 were applied to tissues incubated with C1, C2, and C3 probes, respectively. Nuclei were stained with DAPI, and tissues were mounted with Prolong Gold Antifade mounting solution (Thermofisher Scientific). Slides were stored at 4°C, and tissues were visualized using a Zeiss LSM880 confocal laser scanning microscope at 20–63 x magnification. Images were cropped and labeled using the Zen 2.3 lite application. Cellular outlines of interest were created based on the staining from the channels and outlined using Photoshop (Adobe). We identified infected cell types based on cells that contained both the FISH signal from the VA1 probe and the cell marker probe in the same plane, but subcellular colocalization of the VA1 and cell marker probes was not required as the host and viral RNA can segregate into different locations within a cell (66, 67).

### Strand-specific *in situ* hybridization

Strand-specific RNAscope probes (ACDBio) were designed against the VA1 genome and to not cross-react with other astroviruses, including murine astrovirus. A positive-sense probe was designed against nucleotide positions 2,980–4,024 of the VA1 genome (ORF1b; reference genome accession number NC_013060.1). A negative-sense probe was designed to a different region of the VA1 genome, against nucleotides 4,369–5,361 (ORF2; reference genome accession number NC_013060.1).

To confirm strand specificity, DNA fragments of each target region were generated by PCR, using the VA1 genome encoded in a plasmid as a template. Forward primers containing the T7 promoter were used to generate PCR amplicons with T7 oriented 5′ to the target sequence, in either positive or negative sense (Fig. S3A). The following primer combinations were used: ORF1b + RNA (Forward 5′-TAATACGACTCACTATAGGGTGGAAATTTGCAATGTCAGTGC-3′, Reverse 5′-CCCTCCAAAGCCTATCCAG-3′), ORF1b -RNA (Forward 5′-TAATACGACTCACTATAGGCCCTCCAAAGCCTATCCAG-3′, Reverse 5′-GTGGAAATTTGCAATGTCAGTGC-3′), ORF2 +RNA (Forward 5′-TAATACGACTCACTATAGGCCAAGGCAGCGGCAAAGC-3′, Reverse 5′-CCATTCACTAGAGTCGTGGC-3′) ORF2 -RNA (Forward 5′-TAATACGACTCACTATAGGCCATTCACTAGAGTCGTGGC-3′, Reverse 5′-CCAAGGCAGCGGCAAAGC-3′). PCR fragments were generated using PfuUltra II Fusion High-fidelity DNA Polymerase (Agilent), and PCR products were purified using a PCR purification kit (QIAquick PCR purification kit; Qiagen). BHK-21 cells constitutively expressing T7 were grown on chambered slides and were then transfected with the DNA products using Lipofectamine 3000, following the vendor’s protocol (Invitrogen). The cells were then fixed using 4% PFA 48 hours after transfection and dehydrated in a series of dehydration steps with increasing concentrations of ethanol for storage. The cells were then rehydrated and treated with hydrogen peroxide and RNAscope Protease III using the vendor’s protocol. The cells were then stained using the same protocol as described for FISH.

Caco-2 cells were infected with VA1 and an MOI of 1, and 48 hours after inoculation, they were fixed using the same protocol mentioned above for BHK-21 cells. The mouse tissue was also stained using the strand-specific probes with the FISH protocol.

### Statistical analysis

Prism 10.2 (GraphPad) was used for data analysis and graphing. Comparisons of mouse weights were performed using mixed-effects models. Comparisons between heart and liver RNA viral loads and titers were done using Kruskal Wallis testing, and *post-hoc* testing was done to identify significant pairwise comparisons. The number of CD68-positive cells from infected hearts were compared to those of mock-infected hearts using a nested *t*-test within a mixed-effects model. We confirmed that there was no statistical difference between mice within the mock- or VA1-infected groups. To compare viral RNA loads from immunodeficient mice, Kruskal–Wallis testing was used with *post-hoc* testing to identify significant pairwise comparisons. Adjusted *P* values ≤ 0.05 were considered significant.

## RESULTS

### VA1 RNA is detectable in murine heart tissue up to 21 days post-inoculation

**Fig 1:**
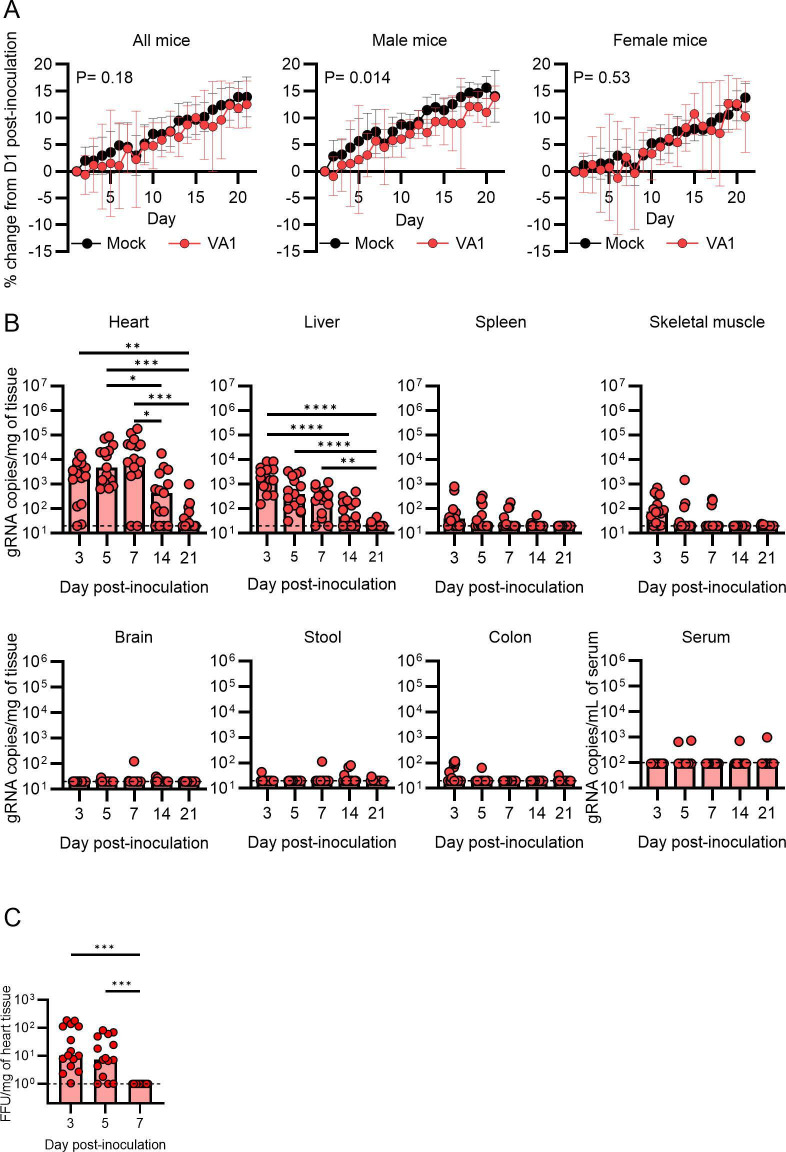
Inoculation of VA1 in wild-type A/J mice results in accumulation of viral RNA in the heart tissue. Wild-type A/J mice were intraperitoneally inoculated with VA1. (**A**) Male but not female mice had reduced weight gain following inoculation with VA1 compared to sex-matched mock-infected mice. (**B**) Over the course of 21 days post-inoculation, the viral RNA load was measured from tissues. The highest viral RNA loads were in the heart, peaking 7 days post-inoculation but remained detectable in some mice at day 21. Viral RNA was also detected from the liver, spleen, and skeletal muscle, while other tissues had sporadic detection of VA1 RNA. The dashed line indicates the limit of detection. (**C**) Viral infectious titers from heart homogenates collected from VA1-inoculated mice were measured by a focus-forming assay. Infectious virus could be detected 3 and 5 days post-inoculation, while it was undetectable on day 7. The dashed line represents the limit of detection. For (**B**) and (**C**), * denotes *P* < 0.05, **P ≤ 0.01, and ***P ≤ 0.001.

We first determined whether VA1 could infect and cause disease in wildtype (WT) mice. Five-week-old A/J mice were inoculated by intraperitoneal injection with 1.5 × 10^7^ focus-forming units of VA1 or mock-infected. Mice were monitored for development of symptoms, weighed daily, and were sacrificed on different days up to 21 days post inoculation (p.i.). No deaths occurred, and the mice did not display any clinical signs of illness (hunched posture, lethargy, ruffled fur, decreased activity, seizures, or paralysis). Mock-infected mice had a median weight gain of 15% during the observation period. In aggregate, VA1-infected mice also gained weight at a similar rate over 21 days compared to mock-inoculated mice (median gain 13%, mixed-effects model *F*[1,97]= 1.84, *P* = 0.18; Fig. 1A). When comparing the weight changes by sex, we noted a significant difference (Fig. 1A). Male mice had lower weight gains relative to mock-inoculated mice (mixed-effects model *F*[1,49]= 6.5, *P* = 0.014), while there was no difference in female mice (mixed-effects model *F*[1,46]= 0.42, *P* = 0.53). 

We next determined the kinetics of VA1 RNA over time and whether inoculation resulted in viral persistence in any particular tissues. Tissues were harvested from mice sacrificed on days 3, 5, 7, 14, and 21 p.i., and viral RNA was measured from the brain, brainstem, heart, lung, liver, kidney, spleen, ileum, descending colon, skeletal muscle, mesenteric lymph node, stool, and serum. Remarkably, the highest viral titer was detected in the heart, with >10^3^ gRNA copies per milligram of tissue (copies/mg) at day 3, peaking at 10^5^ copies/mg by day 7, and some mice remained RT-PCR positive at 21 days p.i. (Fig. 1B). This remained consistent among all experiments with both sexes, with only a small subset of one to two of mice out of 15 testing negative from the heart tissue at each time point in the first 7 days after inoculation. Several other organs had consistently detectable virus, including the liver, spleen, and skeletal muscle, including mice that were negative from the heart tissue (Fig. 1B). In particular, the livers had viral titers of ~10^3^ copies/mg on day 3 and decreased to near or below the limit of detection by day 21. In other tissues, VA1 RNA was infrequently detected and at low quantities (Fig. 1B ; Fig. S1), including the GI tract, serum, and central nervous system.

We also determined whether infectious virus could be recovered from the heart tissue following IP injection. Infectious titers of up to 10^2^ FFU/mg of the tissue were detected from heart tissue on days 3 and 5 p.i., but no infectious virus was detectable at day 7 (Fig. 1C). These results demonstrate that infectious particles are present and recoverable from the heart tissue after IP inoculation into a different body site.

### VA1 is tropic to heart tissue in multiple mouse strains

**Fig 2:**
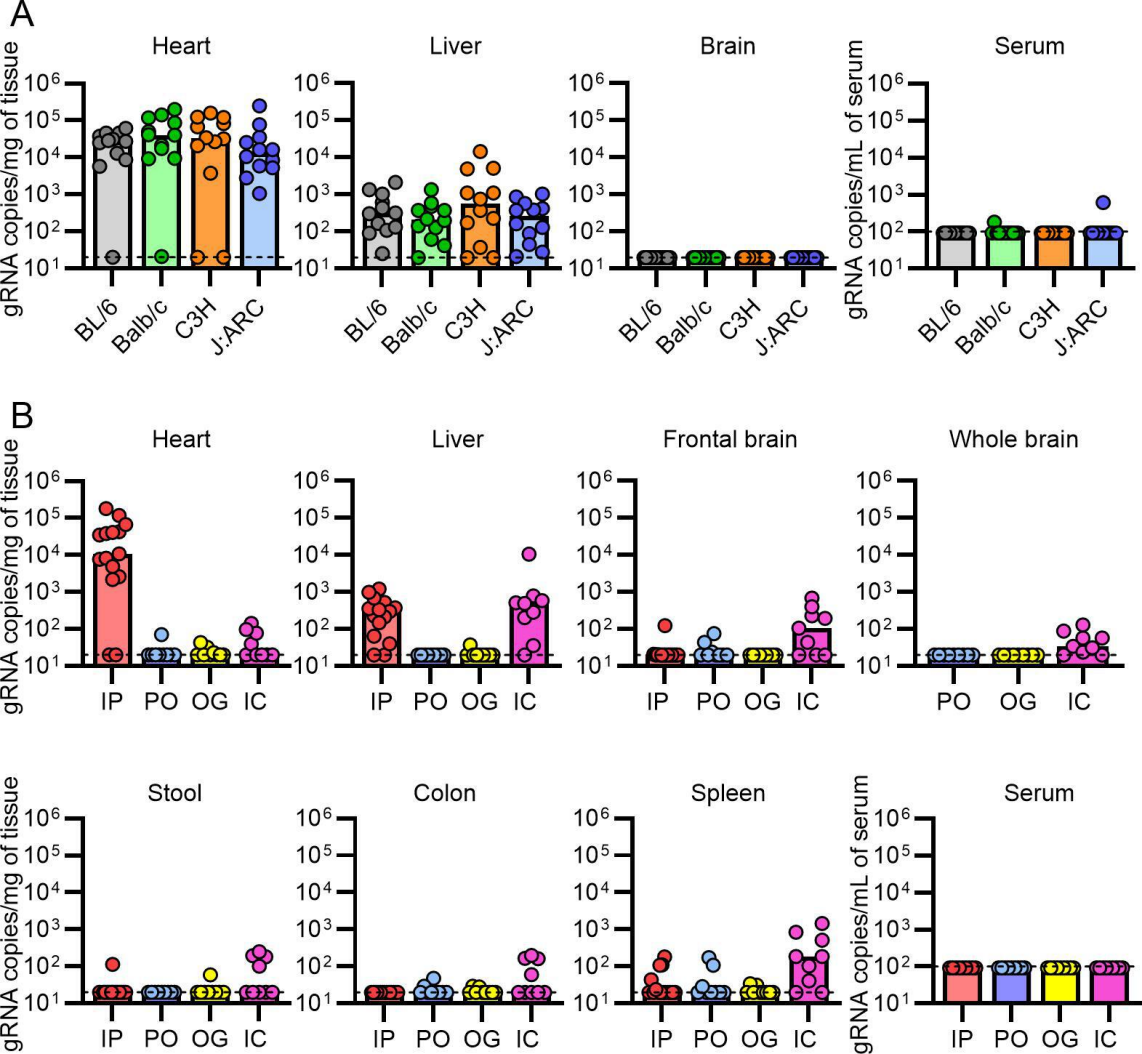
VA1 is cardiotropic in other mouse strains, while inoculation by other routes does not result in significant cardiac infection. Commonly studied mouse strains (C57BL/6 [BL/6], Balb/c, C3H/HeJ, and J:ARC Swiss Outbred) were intraperitoneally inoculated with VA1, and viral load was measured by qRT-PCR 7 days post-inoculation (**A**) Like A/J mice, other strains of mice have a significant viral load in the heart and liver tissue, while there was only sporadic detection in the serum and brain tissue. (**B**) Different routes of inoculation were tested in wild-type A/J mice, including intraperitoneal (IP; data from Fig. 1B, per os (PO), oral gavage (OG), and intracranial (IC) inoculation. Viral RNA loads were measured 7 days after inoculation. PO and OG inoculation did not result in significant infection of mice, including in the heart tissue compared to IP inoculation. IC inoculation resulted in detection of low quantities of viral RNA from the brain tissue, including whole or frontal brain tissue, but was several logs lower than what can be detected in the heart tissue from IP inoculation. Viral RNA was also detected from the liver and spleen, while low quantities were detected from the heart. For all graphs, the dashed line is the limit of detection.

For some viruses, such as influenza and ebolaviruses, different mouse strains can have different capacities to support viral infection (68, 69). We used the A/J mouse genetic background to initially characterize the viral kinetics, which is also commonly used to model myocarditis (70). We tested other strains of mice to determine if VA1 tropisms and RNA loads were dependent on the genetic background. We inoculated VA1 in three commonly studied inbred strains, including C57BL/6, Balb/c, and C3H/HeJ, and a Swiss outbred strain, J:ARC. All strains demonstrated similar viral loads in the heart tissue compared to A/J mice (Fig. 1B and 2A). Like the A/J mice, the other mouse strains also had detectable viral RNA in the liver tissue, while the virus was rarely detected from the brain and serum (Fig. 2A). No deaths occurred, and there were no signs of illness. These results further demonstrate VA1’s cardiotropism in multiple mouse strains.

### Inoculation of mice by different routes does not result in similar cardiac viral RNA loads compared to intraperitoneal inoculation

We also assessed the impact of other routes of inoculation on the tropism of VA1. The fecal-oral route is one of the primary routes of transmission by astroviruses (4–8). Recently, the salivary tract has also been reported as a key site of viral replication for astroviruses and other enteric viruses (*per os* (PO) route) (71). We tested these routes separately by inoculating A/J mice by oral gastric lavage (OG) and by pipetting the inoculum into the mouths of the mice to test the PO route. Virus was undetectable in most mice on day 7, with only a small subset having a very low viral load in any tissue, including the heart, and no evidence of viremia (Fig. 2B). The virus was undetectable in most mice from the GI tract and stool using either PO or OG routes (Fig. 2B).

Given the role of VA1 in causing encephalitis, we also tested the intracranial (IC) route of inoculation. The murine blood-brain barrier may prevent dissemination of the virus to the central nervous system, and the IC route would bypass this potential limitation. Inoculation of mice by IC route resulted in detectable but low copy numbers from the frontal brain tissue and whole brain (Fig. 2B). However, these copy numbers were several logs lower than what we observed in the heart tissue by IP inoculation, despite direct injection into the brain (Fig. 2B), suggesting that replication, if it did occur, is limited. While the virus was not detectable in serum on day 7, we did detect the virus in the liver and spleen (Fig. 2B). These results are consistent with dissemination of the virus out of the central nervous system and transportation to these locations, likely mediated by lymphatics, blood, or circulating immune surveillance cells. While this could reflect some limited viral replication, it likely represents immune clearance as the liver and spleen are important organs that mediate this function (72).

### Intracellular VA1 capsid and RNA are detected in heart tissue, with RNA localizing to cardiomyocytes and endocardial cells

**Fig 3:**
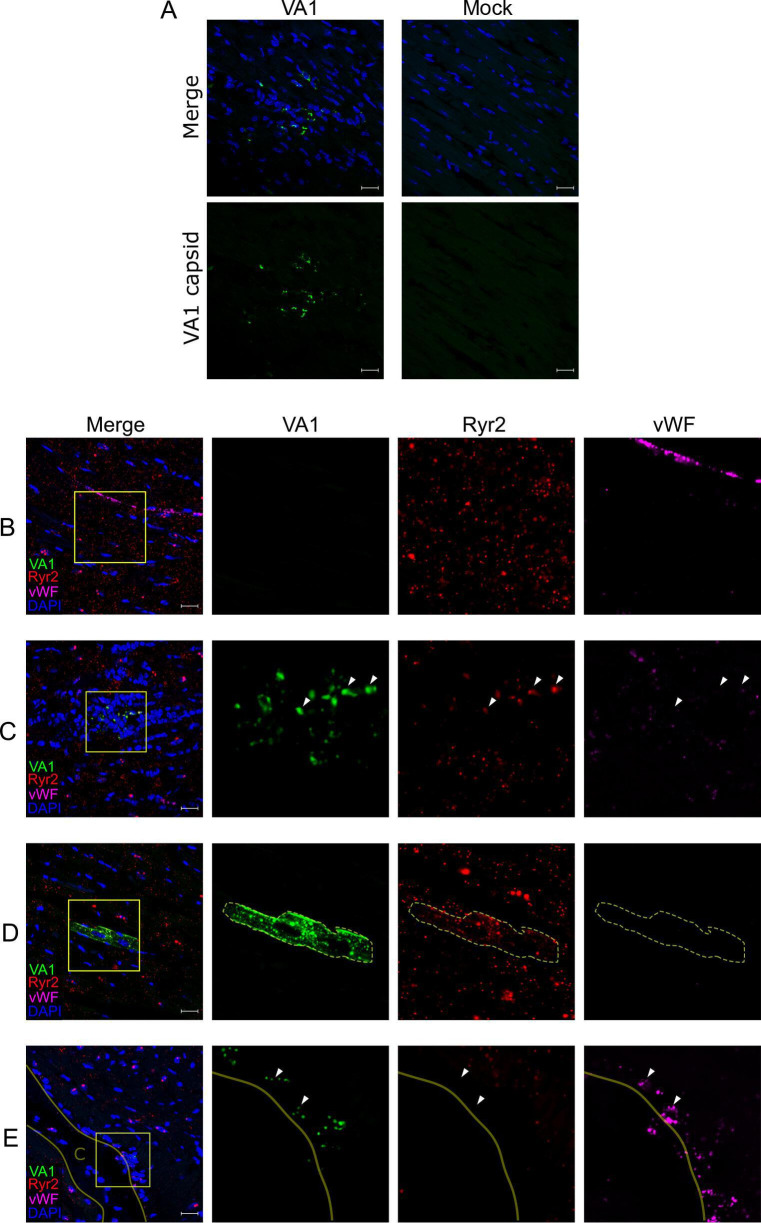
VA1 RNA intracellularly localizes to cardiac myocytes and endocardial cells in wild-type A/J mice. (**A**) Immunofluorescence staining of VA1- or mock-inoculated mouse hearts. Tissues were processed for immunofluorescence and stained with an antibody to the VA1 capsid (green) and counterstained with DAPI. VA1-infected mice had positive punctate signals in regions with increased nuclei. No staining was observed in mock-inoculated mice. (**B–E**) A fluorescent *in situ* hybridization (FISH) assay using probes specific to VA1 open reading frame 2 (ORF2; green) was developed and imaged by confocal microscopy. After 7 days post-inoculation, the heart tissue was obtained and stained with probes to VA1 and to host markers for cardiac cell types including cardiomyocytes (Ryr2 probe; red) and endothelial cells (vWF probe; magenta). Merged fluorescence images are shown with counterstaining of nuclei (performed with DAPI). Boxes demonstrate areas of interest that were further magnified to demonstrate the signal from individual fluorescent channels. Outlines indicate cellular borders of cells containing VA1 RNA. White triangles highlight further areas of staining by the VA1 and host marker probes (**B**) No staining for VA1 was detected in mock-infected mouse hearts. (**C–E**) VA1-infected hearts stain positive for detectable VA1 colocalizing with (**C**) within foci of dense cellular infiltrates and colocalizing with Ryr2, (**D**) cardiomyocytes expressing Ryr2 without cellular infiltrates, and (**E**) within a region of endocardial cells expressing vWF. The solid line and the label C denote the open space of the heart chamber. Scale bars represent 20 µm.

Based on the high abundance of viral RNA in heart tissue, we next determined if we could detect VA1 intracellularly. Detection of intracellular VA1 would further validate that VA1 has entered cells and is not transiently passaging through the tissue or extracellularly attached to cells. We first used an antibody to the VA1 capsid in immunofluorescence. We found that VA1-infected mice had areas with positive staining with punctate signals but unclear cellular borders (Fig. 3A). These regions appeared to have many nuclei present, as indicated by the DAPI staining (Fig. 3A). This result further validates that VA1 infects cells of the murine heart and results in translation of the viral capsid.

To complement the positive intracellular capsid staining results, we developed a VA1 fluorescent *in situ* hybridization assay with confocal microscopy to detect intracellular VA1 RNA and to be used in multiplex with other probes to cellular markers. VA1-specific probes were synthesized to the region of open reading frame 2 (ORF2). This region encodes the virus capsid precursor protein and is predicted to be present in both genomic and subgenomic RNA strands (62, 63). Robust FISH staining was present in VA1-infected Caco-2 cells and was absent in mock-infected cells, validating our probe specificity (Fig. S2). We next performed multiplexed FISH on the heart tissue from VA1-infected and mock-infected WT A/J mice to determine the cellular tropisms. Each tissue was co-stained with the VA1 probe and with markers of cardiac myocytes (ryanodine receptor 2; Ryr2) and endothelial cells (von Willebrand Factor; vWF) and then imaged using confocal microscopy. We examined the tissue for cells coexpressing both VA1 RNA and cell-type markers in the same visual plane to determine the cell types that support viral replication. No signal was detected in the hearts of mock-infected mice, confirming the specificity of the VA1 probe in tissues (Fig. 3B). Expected staining of the cardiac myocytes by Ryr2 and endothelial cells by vWF was detected (Fig. 3B).  In VA1-inoculated mice, we detected intracellular viral RNA in cardiac cells, with the majority being myocytes, as indicated by the localization of Ryr2 and VA1 probes in the same cells (Fig. 3C and D). Two types of staining patterns were observed for VA1. First, we noted punctate co-staining of both the VA1 and Ryr2 probes among clusters of densely packed nuclei without clear cellular borders (Fig. 3C). This finding was consistent with the staining pattern from immunofluorescence to the viral capsid, which contained many nuclei based on DAPI staining (Fig. 3A). These results suggest the possibility that the increase nuclei is due to an inflammatory response that localizes to VA1-infected cardiomyocytes. In the second staining pattern, the probe localized to Ryr2-positive cells, suggestive of cardiac myocytes, without any apparent changes to cellular architecture with most of the cell positive for the VA1 probe (Fig. 3D). These cells did not have any adjacent inflammatory infiltrates (Fig. 3D).

Aside from myocytes, endothelial lineages of cells are common cardiac cell types (73). We also detected the VA1 signal in regions that were expressing vWF and were negative for Ryr2 along the ventricular wall, suggestive of possible endocardial infection (Fig. 3E). The positive VA1 staining was associated with infiltrating cells, consistent with the inflammatory foci that we hypothesized for the VA1-infected cardiac myocytes (Fig. 3C and E). Together, these results clearly demonstrate that VA1 infects cells of the heart (Fig. 3C through E); is associated with increased nuclei, suggesting an inflammatory response; and is not transiently passing through heart tissue or extracellularly attached.

In liver tissue, the *in situ* hybridization stains for viral RNA were negative (results not shown). This suggests greater sensitivity of qRT-PCR compared to FISH and precludes the interpretation of whether infection or transient passage of VA1 occurs in the liver tissue.

### VA1 negative-strand RNA is detectable in heart tissue

**Fig 4:**
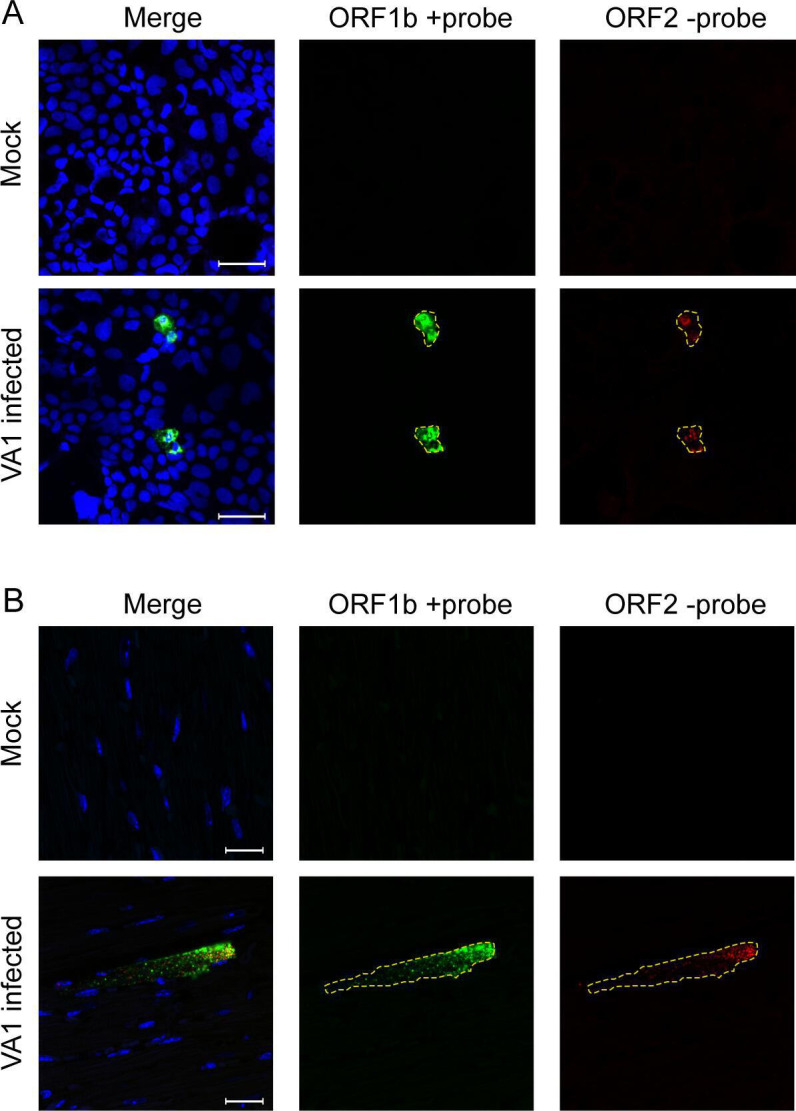
Detection of negative-strand RNA from heart tissue (**A**) Strand-specific FISH staining of Caco-2 cells that were either mock-inoculated or -infected with VA1. The cells were stained with positive and negative sense probes to VA1 48 hours after inoculation. Both RNA strands were detected only in VA1-inoculated cells, consistent with infection and active replication. (**B**) Mock- or VA1-inoculated mice were stained with the strand-specific FISH probes. VA1-inoculated mouse hearts had both positive and negative sense strands being detected, demonstrating that active viral replication was occurring in cardiac tissue. Scale bars represent 20 µm.

Detection of infectious viral particles, intracellular viral RNA, and protein is suggestive that infection and initiation of the viral lifecycle occur in heart tissue. To definitively demonstrate viral replication is occurring in heart tissue, we sought to detect an RNA intermediate produced during transcription. VA1 is a positive-sense, single-stranded RNA virus, and negative-sense RNA is only synthesized during replication (74). We developed strand-specific probes for use in FISH, with a positive-sense probe that targeted a region of ORF1b and a negative-sense probe that targeted a region of ORF2. We first confirmed strand specificity of the probes to positive- and negative-sense strands (Fig. S3A and B). PCR was used to generate DNA fragments encoding the target region for the probes (Fig. S3A). The PCR products encoded a T7 promoter that was orientated in order to transcribe the target sequence in either the positive or negative sense, allowing confirmation of strand specificity (Fig. S3A). The cDNA was used to transfect BHK-21 cells expressing T7 polymerase. Our positive-sense probe (ORF1b + probe) only resulted in fluorescent signal when the ORF1b positive-sense strand was present; likewise, the negative-sense probe (ORF2 -probe) only detected ORF2 negative-sense RNA (Fig. S3B). We next used both positive and negative sense probes in mock- or VA1 infected Caco-2 cells (Fig. 4A). The fluorescence signal was detected in VA1-infected Caco-2 cells, with more abundant and intense signals from the positive-sense probe (Fig. 4A). No signal from either probe was detected in mock-infected cells (Fig. 4A). Using these probes on murine cardiac tissue, we were able to detect both positive and negative sense RNA within cells of VA1-inoculated mice, demonstrating active replication is occurring in VA1-infected cells (Fig. 4B). No signal was detected in mock-infected mice (Fig. 4B).

### Identification of histological features consistent with viral myocarditis

**Fig 5:**
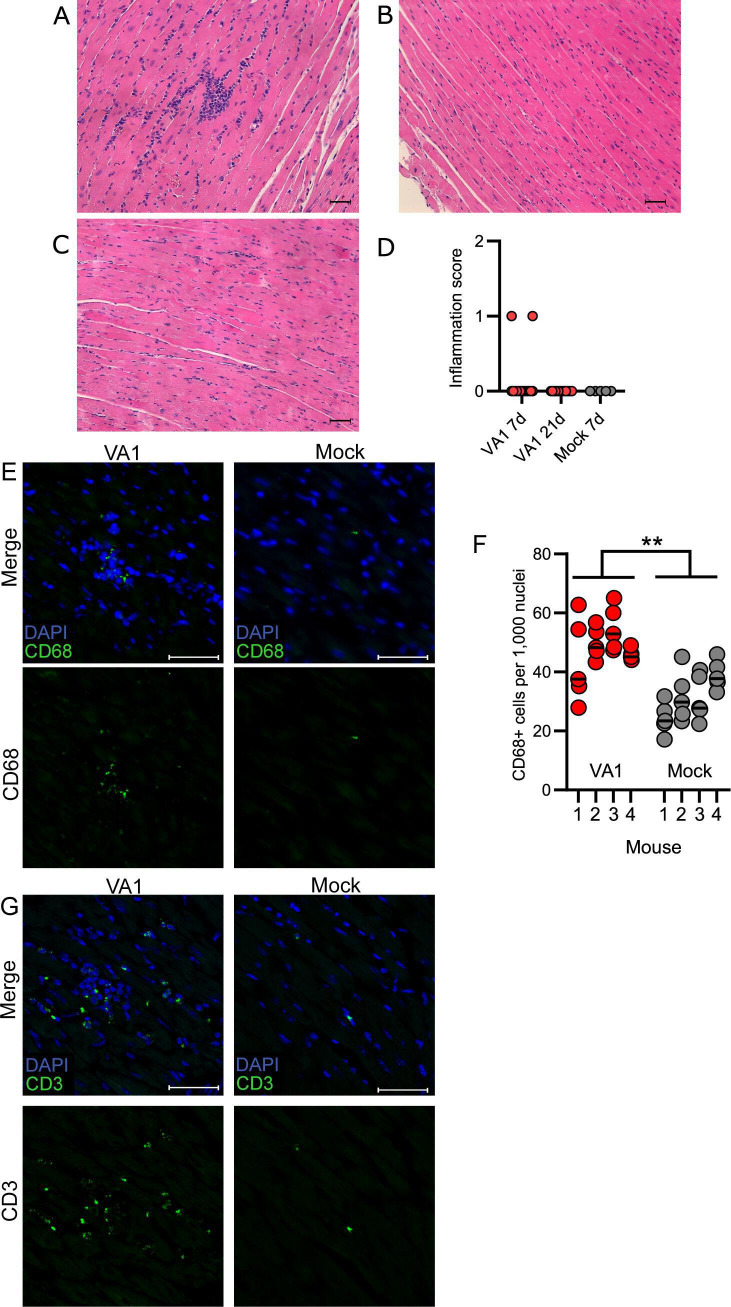
Histological findings consistent with myocarditis are present in VA1-infected heart tissue. Heart tissue histology was analyzed by hematoxylin and eosin staining, with representative images shown. (**A**) Inflammatory infiltrates were present in some heart tissue on day 7, but (**B**) absent at day 21 or (**C**) in mock-inoculated mouse hearts. Scale bars represent 50 µm. (**D**) The presence of focal infiltrates was scored, and they were only present in hearts on day 7 post-inoculation. (**E**) Immunofluorescence was used to stain tissues for CD68, a marker of macrophages and other monocytes. Cells comprising the cellular infiltrates present in VA1-infected heart tissue were CD68-positive. Scale bars represent 20 µm. (**F**) CD68-positive cells were counted in representative tissue regions that lacked foci of cellular infiltrates. A significant increase in CD68-positive cells was identified in VA1-infected (mean 48.5 CD68 +cells per 1,000 nuclei) compared to mock-infected hearts (mean 31.64 CD68 +cells per 1,000 nuclei; nested T test t (6)= 4.35 *P* = 0.005). ** represents *P* ≤ 0.01. A total of four mice in each group were analyzed with five representative sections from each mouse used for counting per mouse. Horizontal lines represent the median value. (**G**) Immunofluorescent staining for CD3, a marker of T cells. As with CD68, many of the cells with a focus of infiltrating cells were CD3-positive and were absent in mock-infected mice. Scale bars are 20 µm.

Given the detection of viral RNA in heart tissue and increased nuclei from immunofluorescence and FISH, we next determined if infection resulted in histological evidence of inflammation by examining hearts collected on day 7 and 21. Hematoxylin and eosin (H&E)-stained sections showed focal interstitial clusters of lymphocytes within the myocardium (Fig. 5A). The cellular infiltrates were predominantly mononuclear, lymphocytic, and were sporadically present, consistent with other animal models of viral myocarditis and in human myocarditis (Fig. 5A) (70, 75, 76). Definitive cardiomyocyte damage was not observed in any cases. Analysis of the pericardium did not demonstrate any clear abnormalities. None of these findings were identified in mock-infected mouse tissue (Fig. 5C). Analysis of heart tissue 21 days p.i. did not reveal any evidence of inflammatory infiltrates or other abnormalities of heart tissue (Fig. 5B). When examining single-tissue sections for each mouse, 2 of the 15 VA1-infected mice had foci of infiltrating immune cells at day 7 p.i., suggesting a mild myocarditis phenotype (Fig. 5D).

We next stained the tissue for cellular markers at day 7 p.i. to determine the cellular types involved in the foci and whether other more subtle histological changes could be identified. We first stained the hearts using CD45 (PTPRC), a marker of nucleated hematopoietic cells (Fig. S4) (77). Robust staining of CD45 was detected from the cells in the foci of infiltrates, demonstrating their hematopoietic origin (Fig. S4). We next stained for macrophages (CD68) and T cells (CD3) as these cells are commonly involved in the pathogenesis of viral myocarditis and contribute to inflammatory infiltrates (78). When staining for CD68, we detected that a proportion of cells in the foci were positive, demonstrating that some of the cells within the foci are macrophages (Fig. 5E). In addition, we also noticed CD68-positive cells to be present diffusely through the heart tissue. When comparing VA1-infected to mock-infected hearts in areas without infiltrating foci, there was an approximate 50% increase in CD68-positive cells (nested *t*-test t (6) = 4.35 P =0.005; Fig. 5F). We also detected that a significant proportion of cells in the foci were positive for CD3, consistent with lymphocytes as a cell population in the foci (Fig. 5G). CD3-positive cells were not detected in a significant quantity outside of the foci to allow for quantification. Taken together, these results demonstrate that macrophages and T cells are involved in the immune response to VA1 infection in the heart, consistent with other models of viral myocarditis (79). It also suggests that there is diffuse mobilization of macrophages into the heart tissue, based on the increased number of CD68-positive cells.

Given the detectable viral loads in the liver, we also stained the liver tissue on days 3 and 7 p.i.. The liver tissue did not demonstrate evidence of inflammation on histology (data not shown).

### Immunodeficient mice are viremic and have increased viral RNA loads in heart tissue

**Fig 6:**
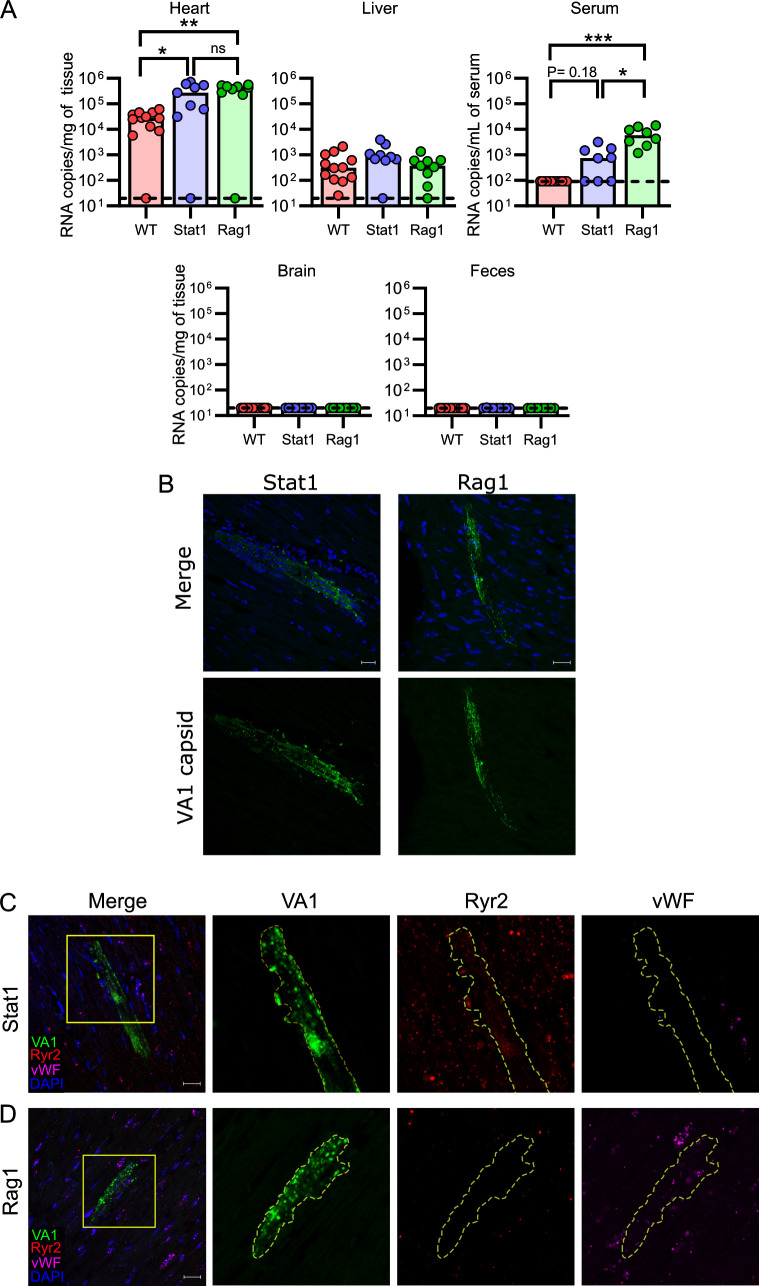
Immunodeficient mice support higher loads of VA1 in the heart and serum. Wild-type (WT) C57BL/6, Rag1 KO, and Stat1 KO mice were inoculated with VA1 by the IP route, and tissues were collected 7 days post-inoculation. (**A**) VA1 copy number detected by qRT-PCR revealed higher quantities of VA1 RNA from the heart tissue and serum of Rag1 KO mice compared to C57BL/6 WT (WT data from Fig. 2). Stat1 KO mice had higher quantities of viral RNA in the heart tissue. *** represents *P* value ≤ 0.001, ** represents *P* value ≤ 0.01, * represents *P* value ≤ 0.05, and ns represents *P* value > 0.05. (**B**) Immunofluorescence for the VA1 capsid in immunodeficient mouse hearts. Both genetic backgrounds had positive staining for the viral capsid, including large cell(s) with morphology consistent with that of cardiac myocytes. (**C–D**) Representative fluorescent *in situ* hybridizations for VA1 (green) in the heart tissue, co-stained for cardiomyocytes (Ryr2 probe; red), and endothelial cells (vWF probe; magenta), with nuclei stained with DAPI (blue). Boxes highlight regions of interest that were further magnified. Scale bars represent 20 µm. Outlines highlight cells in which host markers localize to VA1 probes. (**C**) We detected VA1 RNA in cells expressing Ryr2, demonstrating infection of cardiac myocytes in both Stat1 and Rag1 mice, with a representative infection depicted from a Stat1 mouse. (**D**) In Rag1 KO mice, we also identified VA1-infected cells (green) that were expressing vWF (magenta) but were not expressing Ryr2 (red), suggestive of endothelial cells.

Previous publications have demonstrated roles of the innate and adaptive immune responses during murine astrovirus infection (55, 58, 60). Recombinant activating gene 1 (Rag1) is a gene critical for recombination of B and T cell receptors, and knockout of Rag1 results in mice that are deficient in these cell lineages. Signal transducer and activation of transcription 1 (Stat1) knockout mice have a defect in interferon (IFN) response signaling, resulting in mice insensitive to IFN with reduced capacity to activate antiviral responses. Rag1 and Stat1 KO mice in the C57BL/6 background were inoculated with VA1 by IP inoculation, euthanized on day 7 p.i., and compared to WT C57BL/6 mice. Following inoculation, both Stat1 and Rag1 KO mice showed no clinical signs of infection or mortality. Both immunodeficient genotypes had 10-fold higher titers of virus in heart tissue (>10^5^ gRNA copies/mg) compared to WT (10^4^, Kruskal–Wallis H (3)= 11.6, *P* = 0.003, *post-hoc* testing for Rag1 *P* = 0.006 and Stat1 *P* = 0.02; Fig. 6A). In both immunodeficient backgrounds, the viral capsid could be detected in the heart tissue, with staining in cells consistent with cardiac myocytes (Fig. 6B). Viral RNA was detected in cardiac myocytes, as labeled by Ryr2-positive cells (example of Stat1 KO mouse; Fig. 6C). We also detected in Rag1 KO mice a subset of infected cells that were positive for vWF and negative for Ryr2 staining, consistent with possible endothelial infection (Fig. 6D). Negative sense RNA was also detected in both immunodeficient backgrounds, demonstrating active viral replication (Fig. S5).

Interestingly, immunodeficient mice were viremic (Kruskal–Wallis H (3)= 20.5, *P* < 0.001; Fig. 6A), with 100% of Rag1 (*post-hoc* testing *P* < 0.001) and ~63% of Stat1 KO (*post-hoc* testing *P* = 0.18) being viremic compared to none of the WT mice (Fig. 6A). A statistical difference in the Stat1 KO compared to WT mice becomes apparent if binary positive/negative qRT-PCR results are used (Fisher’s exact test *P* = 0.005). Both cohorts of immunodeficient mice had VA1 RNA present in the liver, while most other organs only had sporadic low-levels of viral RNA, including the brain (Fig. 6A), suggesting no additional tissue tropisms were identified compared to WT mice.

### Inflammatory foci in heart tissue are absent in Rag1 KO mice

**Fig 7:**
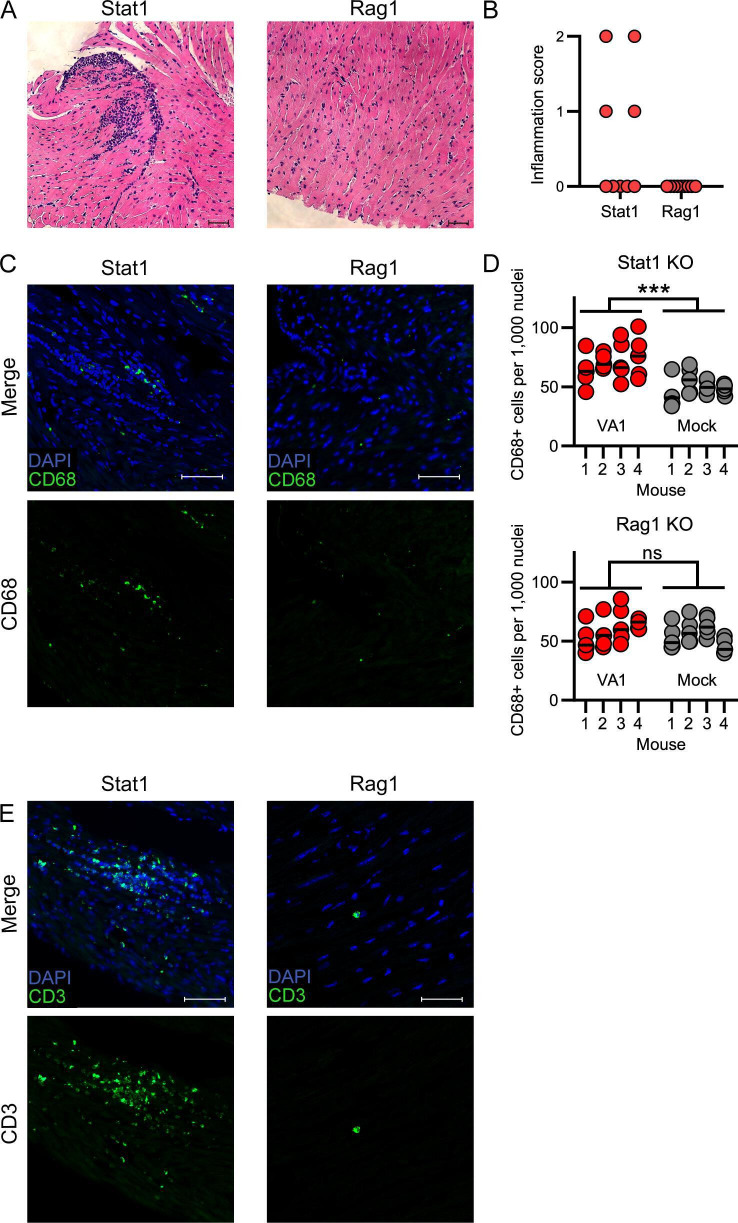
CD68- and CD3-positive cellular infiltrates are present in Stat1 KO but not Rag1 KO mice (**A–B**) Hematoxylin and eosin staining of heart tissue collected from (**A**) Stat1 KO mice shows clusters of cellular infiltration, which are absent in Rag1 KO mice. Scale bars represent 50 µm. (**B**) Quantification of inflammation scores from Stat1 KO and Rag1 KO heart tissue 7 days post-inoculation. Foci were identified in Stat1 KO mice, but none were present in Rag1 KO mice. (**C**) CD68-positive cellular infiltrates are present in Stat1 KO but not Rag1 KO mice, as detected by immunofluorescence. Scale bars represent 50 µm (**D**) In representative tissue regions excluding foci of infiltrating cells, Stat1 (nested T test t (6)= 5.65 *P*< 0.001) but not Rag1 KO mice (nested T test t (6)= 0.85 *P* = 0.43) had significant increases in the number of CD68-positive cells compared to mock-infected mice. Four mice per group were analyzed with five sections counted per mouse. Horizontal lines represent median value. *** represents *P* value ≤ 0.001, ns represents *P* value > 0.05. (**E**) Immunofluorescence of heart tissue for CD3 with a significant number of cells positive for CD3 in the infiltrating foci in Stat1 KO mice. Rare CD3 signal was detected in Rag1 KO mice. Scale bars represent 50 µm.

Histopathological analysis of the heart tissue from immunodeficient mice revealed differences between Stat1 and Rag1 KO mice. On H&E staining, Stat1 KO mice had multiple large cellular infiltrates, while no infiltrates were identified in the heart tissue from Rag1 KO mice (Fig. 7A and B). We next stained the tissue for CD68 and CD3. The foci in Stat1 KO mice contained cells that were either CD68- or CD3-positive (Fig. 7C and E). We also noted a significant increase in the number of CD68-positive cells compared to mock infection in fields without foci in the Stat1 KO mice (nested T test t (6)= 5.65 P< 0.001; Fig. 7D). In Rag1 KO mice, however, there was no increase in the number of CD68-positive cells compared to mock infection (nested T test t (6)= 0.85 *P* = 0.43 ; Fig. 7C and D). Not surprisingly, Rag1 KO mice had very rare staining for CD3, consistent with the rare viability of T cells from this mouse strain and expression of CD3 on other cell types including NKT cells (80, 81). Collectively, these results indicate that both innate and adaptive immune systems are important in the antiviral response to VA1 infection. Infection of both immunodeficient mice results in increases in viral RNA, but only Stat1 KO mice can develop inflammatory foci and have increased number of CD68 +cells in the heart tissue. The absence in Rag1 KO mice would suggest that lymphocytes are important for the formation of the foci and further recruitment of the monocytes/macrophages to heart tissue.

### VA1 can replicate in human cardiac endothelial cells *in vitro*

**Fig 8:**
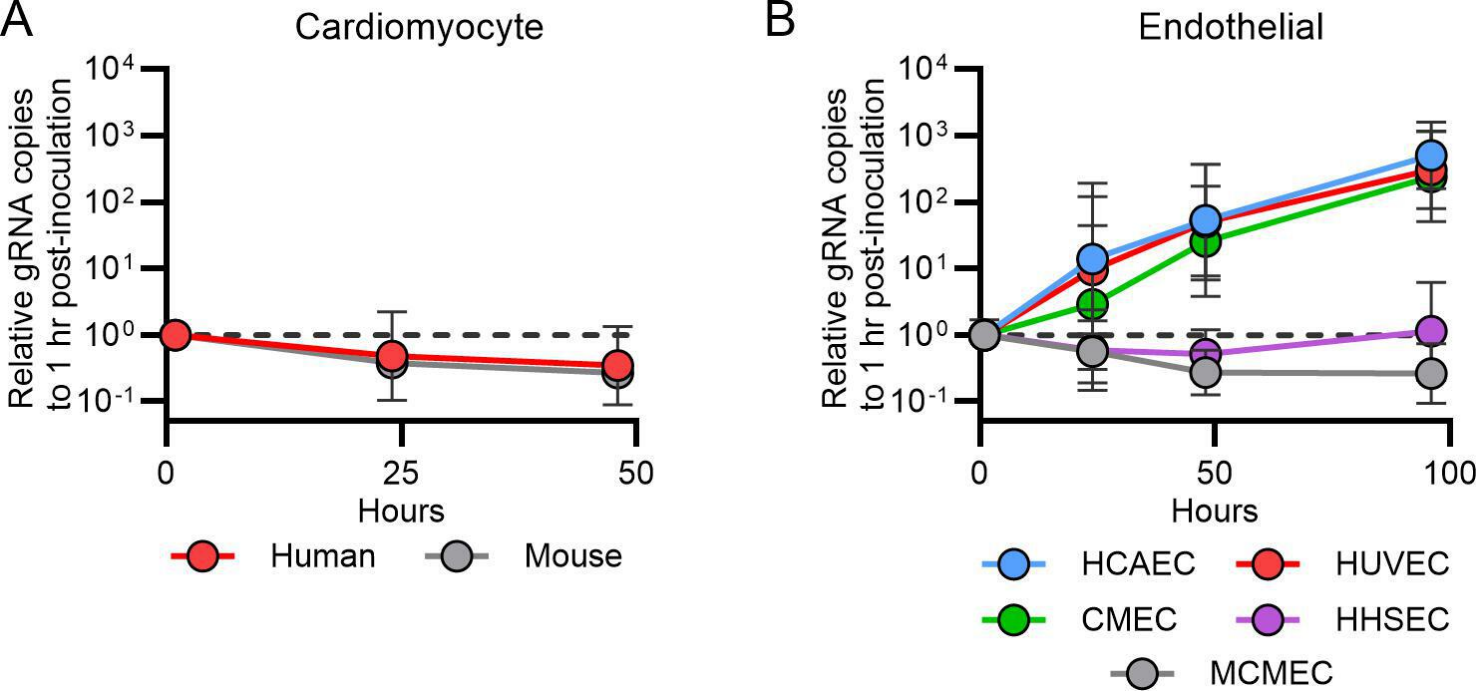
VA1 infects human endothelial-derived cell lines *in vitro.* Growth curves of VA1 using a multiplicity of infection of 3 in (**A**) primary human and mouse cardiac myocytes and (**B**) primary human endothelial cells including microvascular endothelial cells (CMECs), human coronary artery endothelial cells (HCAECs), human umbilical vein endothelial cells (HUVECs), human hepatic sinusoidal endothelial cells (HHSECs), and mouse cardiac endothelial cells (MCMECs). Each data point is normalized to the gRNA copy number present at 1 hour post-inoculation for each cell line, and geometric means are plotted, with error bars representing one geometric standard deviation. The horizontal dashed line represents the relative gRNA copy number at 1 hour post-inoculation.

Based on the tropism we identified *in vivo*, we determined whether VA1 could replicate in primary murine or human cardiac myocytes (Fig. 8A). In multi-step growth curves, there was no increase in VA1 RNA over time (Fig. 8A). These results suggest that primary cardiac myocytes are non-permissive to infection *in vitro*, and additional factors affect the tropism we identified *in vivo*.

The FISH assay also localized VA1 to the endocardium and endothelial cells. Endocardial cells are functionally related to endothelial cells (82). Based on the identification of an endothelial tropism, we tested the capacity of primary endothelial cells to support VA1 replication. We used primary human cardiac microvascular endothelial cells (CMECs), human coronary artery endothelial cells (HCAECs), and primary mouse cardiac microvascular endothelial cells (MCMECs). We also tested two non-cardiac primary human endothelial cell lineages including umbilical vein cells (HUVEC) and hepatic sinusoidal cells (HHSEC). Interestingly, we identified a ~ 100 fold increase in viral RNA in primary human CMEC, HCAEC, and HUVEC cells (Fig. 8B). No significant replication was identified in HHSECs or MCMECs (Fig. 8B). These results demonstrate that VA1 can replicate in human endothelial cells, including those derived from cardiac tissue, and further support the tropism identified from the mouse inoculations.

## DISCUSSION

We describe here the first mouse model of infection using a human-infecting astrovirus species. Our data demonstrate that VA1 RNA accumulates within the hearts of infected mice; localizes intracellularly in myocytes, endothelial, and endocardial cells, resulting in translation of viral capsid; and can trigger an inflammatory response consistent with myocarditis. We further determined that *bona fide* viral replication occurs in heart tissue using a probe specific for negative-sense viral RNA. We also demonstrate the reproducibility of this phenotype across multiple mouse genetic backgrounds, with greater viral loads in immunodeficient mice deficient in either innate or adaptive immunity. In addition, we showed that infectious virus could be recovered from heart tissue. Our results, through a combination of viral culture and molecular-based approaches, establish a causal relationship between astrovirus infection and cardiovascular disease in mice as they are consistent with the criteria established in Koch’s postulates and revised molecular-based guidelines (83, 84).

These findings highlight the importance of animal models of infection as this model revealed a novel cardiovascular tropism. Prior to this study, there was not a clear connection between astrovirus infection and cardiovascular disease. This has led to a paucity of testing and data regarding astrovirus infection of the heart. In few studies, heart tissues from ducks, chickens, or geese infected with astroviruses were positive by PCR (36–38, 40, 85). In addition, human astrovirus was detected from the heart tissue of one human case concerning for encephalitis (39). However, it is unknown if these results reflect the transient presence of viral RNA in the tissue or blood or *bona fide* infection because histological testing for virus was not completed. Rawal et al. reported findings from pigs with neurological disease associated with porcine astrovirus. In one animal, there was histological evidence of myocarditis, but PCR for testing astrovirus from heart tissue was negative (37). The cardiotropism of VA1 suggests that these previously reported findings may not be incidental and could be consistent with cardiovascular infection. Further studies need to be done to more definitively link astrovirus infection to cardiovascular disease in humans.

The murine model of VA1 infection has similarities to other models of viral infection of the cardiovascular system. For VA1, we detected RNA in the heart tissue up to 21 days post-inoculation, with infectious virus detectable on days 3 and 5 (Fig. 1B and C). Similar kinetics of infection have been observed with Coxsackie B3, commonly used in a murine model of viral myocarditis (76). Coxsackie B3 RNA can be detected in the heart tissue up to 90 days post-infection, while infectious viral titers were only detected within the first week after inoculation (76). When both RNA and infectious titers are detectable in the heart tissue, they measured a 10,000-fold difference between RNA and viral titer (76). Likewise with the VA1 model, we also found a 1,000- to 10,000-fold difference between the RNA viral load and titer. The VA1 infection model also demonstrates immune responses that are present in other cardiac inflammatory models. The histological definitions of myocarditis have expanded and now encompass multiple metrics including histological findings of heart infiltrates and quantification of T cells and macrophages (70). We demonstrated cardiac infiltrates in mice infected with VA1, and within these infiltrates, we specifically identified CD3 +T cells and CD68 +macrophages (Fig. 5). When compared to mock-infected mice, VA1-infected mice had a significant increase in CD68 +macrophages, consistent with definitions of myocarditis (Fig. 5) (70). Further epidemiological and experimental studies are needed to determine whether the observed murine cardiotropism translates to cardiovascular diseases in humans and other organisms.  Astroviruses could be a factor in cardiovascular health that has gone unrecognized.

Prior to this study, there were also major limitations for studying astroviruses *in vivo*. For the turkey astrovirus model, it is limited by the lack of turkey-specific reagents and clonal populations of turkeys (1). The mouse model of murine astrovirus is complicated by the frequent colonization of mice by murine astrovirus and the lack of a disease associated with infection (55, 58, 60). Previous attempts at generating an animal model for human astrovirus infection did not result in clear infection of the mice, with only transient detection of viral RNA in the spleen (55). Our model of VA1 infection demonstrates that human VA1 astrovirus can cause cross-species infection. This raises further hypotheses about conserved pathways between humans and mice that are utilized by the virus for the viral lifecycle, including whether the entry receptor(s) for VA1 are the same or different between species. It is also not clear whether the cardiotropism is specific to VA1 or if other human-infecting astroviruses infect heart tissue in mice. It is also unclear if VA1 could be used for inoculation of well-studied organisms, including nonhuman primates, and exhibit the same cardiotropism.

This study provides important immunological insights into the response to human astrovirus infection. We identified increased viral RNA loads in heart tissue and viremia from Rag1 KO mice, supporting the role of the adaptive immune response during infection. We also did not identify any inflammatory foci or increase in CD68 +cells in the Rag1 KO mice. This suggests that formation of the foci and further recruitment of monocytes/macrophages during VA1 infection is mediated by lymphocytes. Lymphocyte-mediated elimination of virally infected cells could be a mechanism that explains the increase in viral RNA. Increased viral loads have been also identified in models of murine astrovirus infection using mice with genetic defects in the adaptive immune response (55, 58). In humans, a majority of the patients with astrovirus-associated encephalitis had defects of adaptive immunity. Some subjects had a primary immunodeficiency including X-linked agammaglobulinemia, while others had acquired immunodeficiencies due to recently receiving chemotherapy or a hematopoietic stem cell transplant (1). Astroviruses could be opportunistic pathogens in humans. The lack of a coordinated lymphocyte response could lead to increased viral replication and viremia, thus promoting invasive diseases. Components of cellular immunity, including cytotoxicity and downstream processes to clear virus enabled by effector T cells, could be important for the immune response and can be further studied. Future work will also determine the role of antibody-mediated neutralization and Fc effector functions.

Our results also suggest the importance for the innate immune response to VA1 infection as we observed higher quantities of VA1 RNA in Stat1 KO mice compared to WT mice. This result is consistent with past results where we have previously shown *in vitro* that VA1 is IFN-sensitive (62, 86). IFN is also important in the models of murine astrovirus infection, where higher quantities of murine astrovirus RNA were detected in IFN-deficient mouse backgrounds (55, 58). We hypothesize that the increase in viral RNA in Stat1 KO mice is due to disruption of IFN signaling, leading to a diminished innate immune response (87). However, we cannot rule out an alternative possibility that Stat1 could be mediating effects through adaptive immunity as Stat1 also has a role in antigen presentation and antibody class switching (88). Nonetheless, the Stat1 KO mice had inflammatory foci containing T cells and increased numbers of macrophages that were also present in the WT mice. These results suggest the Stat1 KO mice are mounting an adaptive immune response to some extent. Future work will dissect the mechanisms behind why Stat1 KO mice have an increase in VA1 RNA, identifying whether it is due to a reduced innate immune response or is mediated by an alternative pathway.

The VA1 infection model revealed novel tissue tropisms of VA1 in cardiomyocytes, endothelial, and endocardial cells. This provides a direct route by which VA1 could cause cardiac injury by mediating an inflammatory response. In addition, VA1 replicates in multiple human endothelial cell lines, including HUVECs. HUVECs, which are derived from the umbilical cord vasculature, are a commonly used *in vitro* system for studying endothelial cells (89). Their capacity to support VA1 infection raises additional hypotheses regarding the role of VA1 infection during pregnancy. To our knowledge, there is currently no data regarding astrovirus infection in maternal or fetal studies, pointing to a gap in astrovirus biology that future studies can address. The endothelial tropism of VA1 has many pathological consequences as other viral infections of endothelial cells promote the expression of proinflammatory cytokines and chemokines (90, 91) that can be now further evaluated *in vitro* and *vivo*. Vascular endothelial cells are known for their ability to present the foreign peptide to circulating immune cells and facilitate transmigration and recruitment of immune cells via chemokine and integrin expression (92). It is possible that during VA1 infection, endothelial cells propagate cellular infiltration and recruitment to sites of infection via presentation of VA1 antigen on MHC complexes. Future studies can further investigate the role of endothelial-mediated antigen presentation as a mechanism of cardiac inflammation.

Inoculation of VA1 into primary cultures of murine myocytes and endothelial cells did not support viral replication. Viral *in vivo* tropisms do not always directly translate to *in vitro* tropisms as decades of work were needed to propagate human norovirus in GI cells and hepatitis C in hepatic cells (93, 94). Furthermore, murine astrovirus has been difficult to cultivate *in vitro* despite clear evidence of infection of the gastrointestinal tract *in vivo* (95, 96). Murine astrovirus is incapable of propagation in three-dimensional enteroids, and there is only around a tenfold increase in viral RNA using air-liquid interface cultures derived from mouse enteroids (95). There are likely additional factors yet to be identified that contribute to the tropisms of astroviruses *in vitro* and *in vivo,* which affect their capacity to replicate in these different models.

Despite the known fecal-oral transmission route and association of gastroenteritis with astroviruses in humans, mice are largely resistant to PO and OG challenge with VA1. A similar result has been published with other human-infecting astroviruses (55). While VA1 infected the heart in IP-inoculated mice, we did not observe significant infection of the GI tract. The factors conferring the resistance of the GI tract are unknown. Mice can be resistant to oral challenge but susceptible to other routes of inoculation with other well-known fecal-orally transmitted viruses in humans, including many enteroviruses (97). Currently, the receptor for VA1 is unknown, so it is unclear whether the entry receptor affects the tropism to the GI tract and therefore susceptibility. In addition, Ingle et al. previously demonstrated that laboratory mice were resistant to infection with murine norovirus when the mice were co-infected with a specific murine astrovirus strain (98). Murine astrovirus infection was associated with elevated IFN-λ, which was hypothesized to prevent infection with norovirus or other viruses (98). Interestingly, in our IP infections of Stat1 KO mice (Fig. 6A), we did not detect any VA1 RNA in the feces. Stat1 is important for the signaling cascade for IFN-λ (99), so these results would not be consistent with the hypothesis that IFN-λ is restricting VA1 infection of the GI tract. In the future, we can further determine how host factors like the entry receptor, IFN-λ, and other confounders like murine astrovirus infection affect the infectability and tropism of VA1 *in vivo*. Beyond the GI tract, in humans, VA1 has been identified from several other tissues, including the respiratory tract. Though fecal-oral is the presumed primary route of transmission for VA1, other routes of inoculation may be possible in this model. Future experiments can rule out the possibility that VA1 is transmitted in any capacity via aerosols or droplets.

The VA1 mouse model does have limitations in modeling astrovirus-associated human diseases. Astroviruses cause encephalitis in humans, but we note the lack of clear evidence of VA1 infection in the central nervous system by either IP or IC routes of inoculation. Other human neurotropic viruses have shown to lack a neurotropism in mice or required adaptation to cause neurological diseases including poliovirus, measles, enterovirus 71, and enterovirus D68 (100–104). We have previously shown in the cell culture that VA1 can infect astrocytes, but neurons are resistant to infection, despite a neuronal tropism being identified from clinical cases (11, 63). In addition, VA1 did not cause significant infection of the GI tract despite the link of astroviruses with gastrointestinal diseases (3) Together, these results suggest there are additional unknown factors that affect the viral tropism *in vitro* and *in vivo*, and they can be further characterized in the future.

In our study, we also used an infectious dose of ~10^7^ FFU per mouse, but the implication of this dose is unclear. In other well-adapted murine models of viral infection, inoculums can range from 10^2^ to 10^3^ infectious units (105, 106). For human and murine astroviruses, the infectious dose has been largely undefined. Challenge studies with humans with human-infecting astroviruses or mice with mouse-infecting astroviruses used defined volumes but did not quantitate the infectious dose (5, 6, 55, 59, 60). One possibility is that there is a difference in human and mouse susceptibility to VA1, and there could be a barrier to infection in mice. However, this barrier is not absolute as our study is the first to demonstrate the replication of a human-infecting astrovirus in mice. It is possible that further adaptation of the virus to mice will result in more robust infection or different tropisms. Future work can focus on serially passaging VA1 in mice to determine if the virus accumulates adaptive mutations that confer a change in tropism or pathogenicity in mice.

Astrovirus infection in humans is very common based on seroprevalence studies. We have established a tractable mouse model of VA1 infection, which will be an important tool to study the pathogenesis of human astrovirus infection *in vivo.* In addition, the model also establishes a previously unappreciated cardiovascular tropism in mice, creating additional hypotheses regarding the role of astroviruses in cardiovascular health. Ultimately, this model enables further understanding of the biology of astroviruses *in vivo* and will allow for future studies in the development of astrovirus-specific therapeutics and vaccines.

## Data Availability

In accordance with open data policies, data generated through this work, including qPCR, mouse and tissue weights, FFU measurements, and images, are publicly available through the Digital Commons Data@Becker repository, DOI: 10.17632/vjdffkxs8z.2

## SUPPLEMENTAL MATERIAL

Figure S1 jvi.00240-25-s0001.tif VA1 RNA in other mouse tissues.

Figure S2 jvi.00240-25-s0002.tif FISH assay confirmatory testing.

Figure S3 jvi.00240-25-s0003.tif Strand specific FISH validation.

Figure S4 jvi.00240-25-s0004.tif CD45 staining.

Figure S5 jvi.00240-25-s0005.tif Negative stand RNA detection from Stat1 and Rag1 KO mice.

Supplemental legends jvi.00240-25-s0006.docx Legends for Fig. S1 to S5.

ASM does not own the copyrights to Supplemental Material that may be linked to, or accessed through, an article. The authors have granted ASM a non-exclusive, world-wide license to publish the Supplemental Material files. Please contact the corresponding author directly for reuse.
