# Supplementary figures and images for: Novel murine model of human astrovirus infection reveals cardiovascular tropism
Source: J Virol. 2025 Apr 30;99(5):e00240-25. doi: 10.1128/jvi.00240-25 (PMC12090817; doi:10.1128/jvi.00240-25)

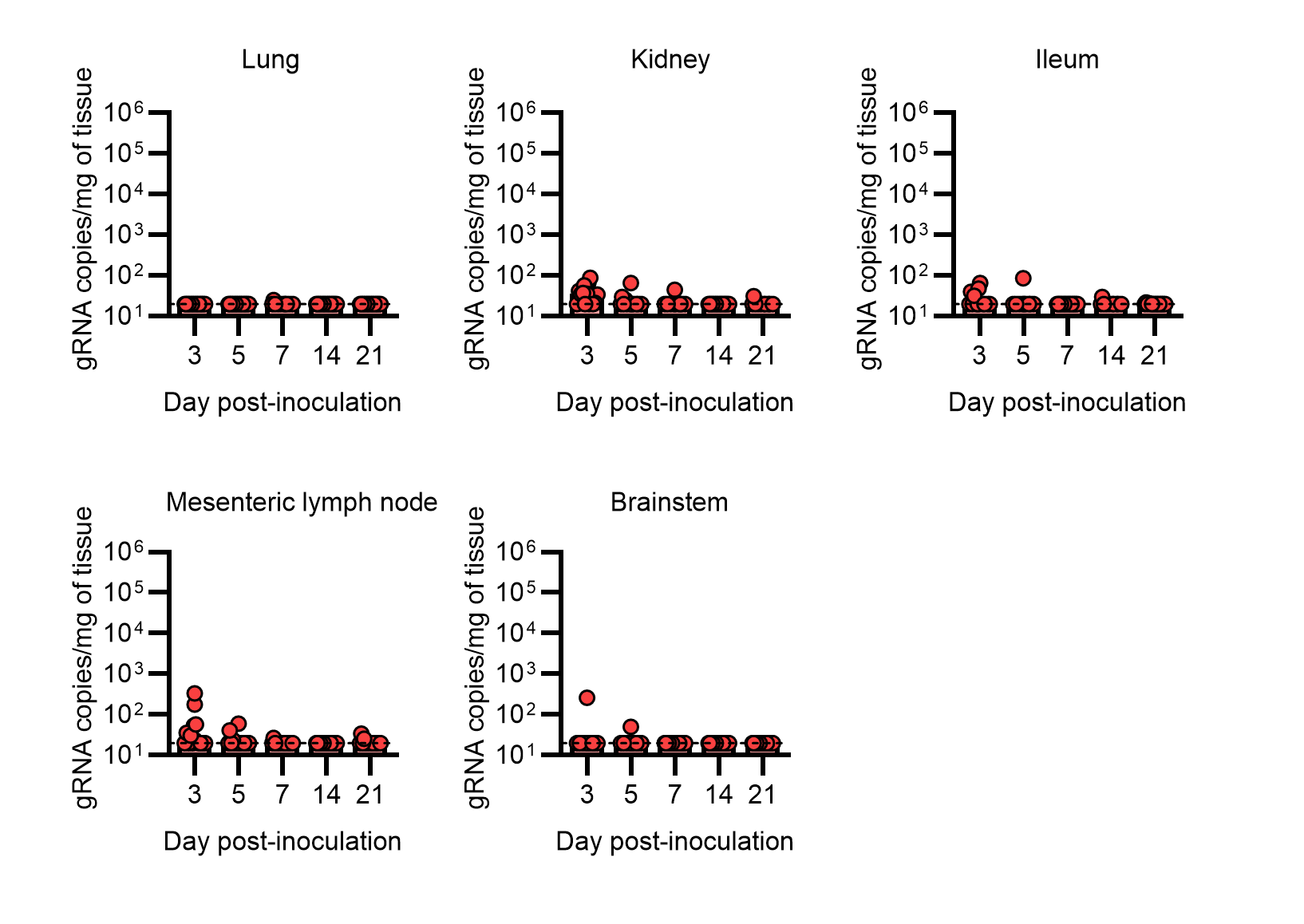

Supplement: Figure S1 — VA1 RNA in other mouse tissues. [file jvi.00240-25-s0001.tif]

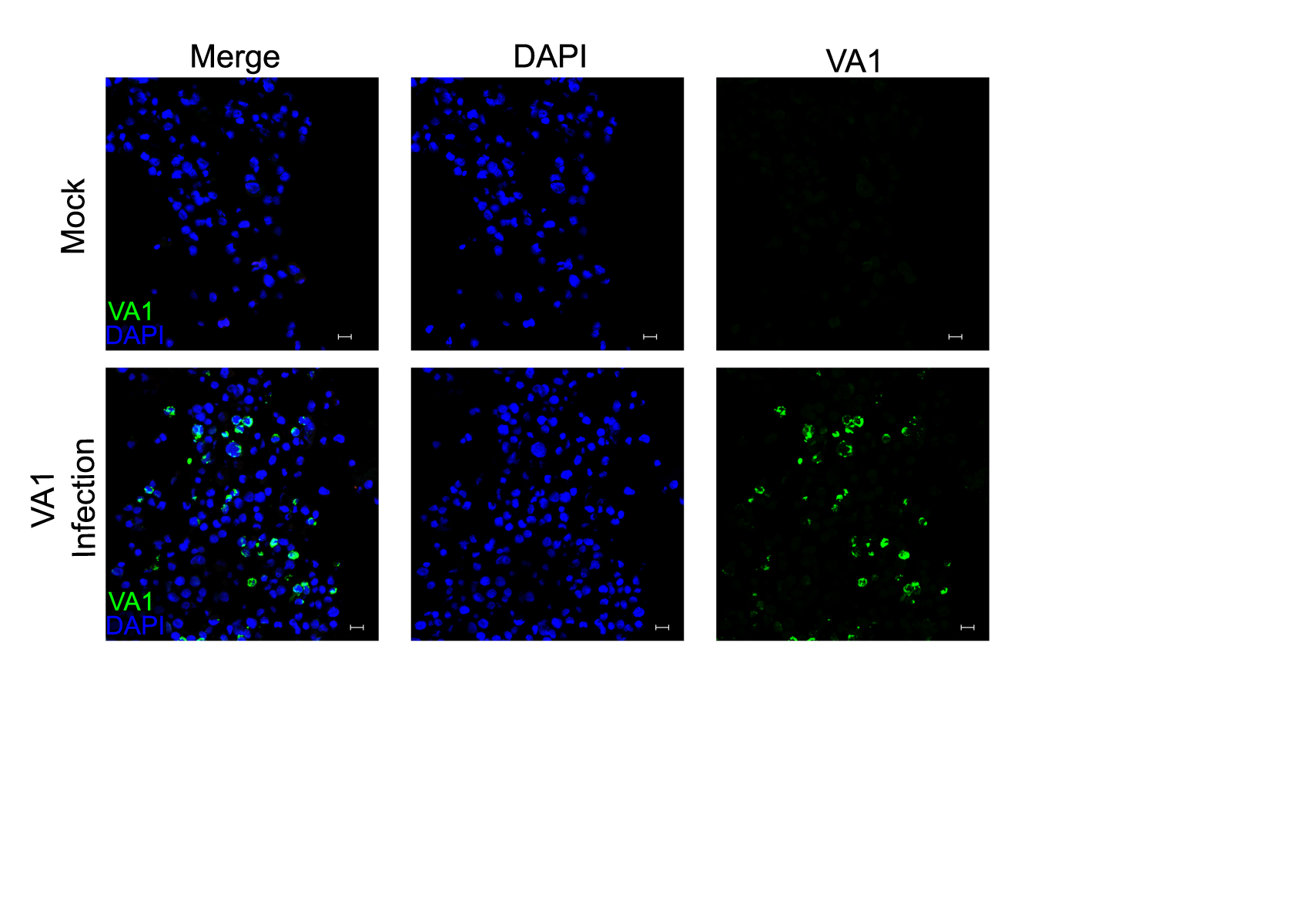

Supplement: Figure S2 — FISH assay confirmatory testing. [file jvi.00240-25-s0002.tif]

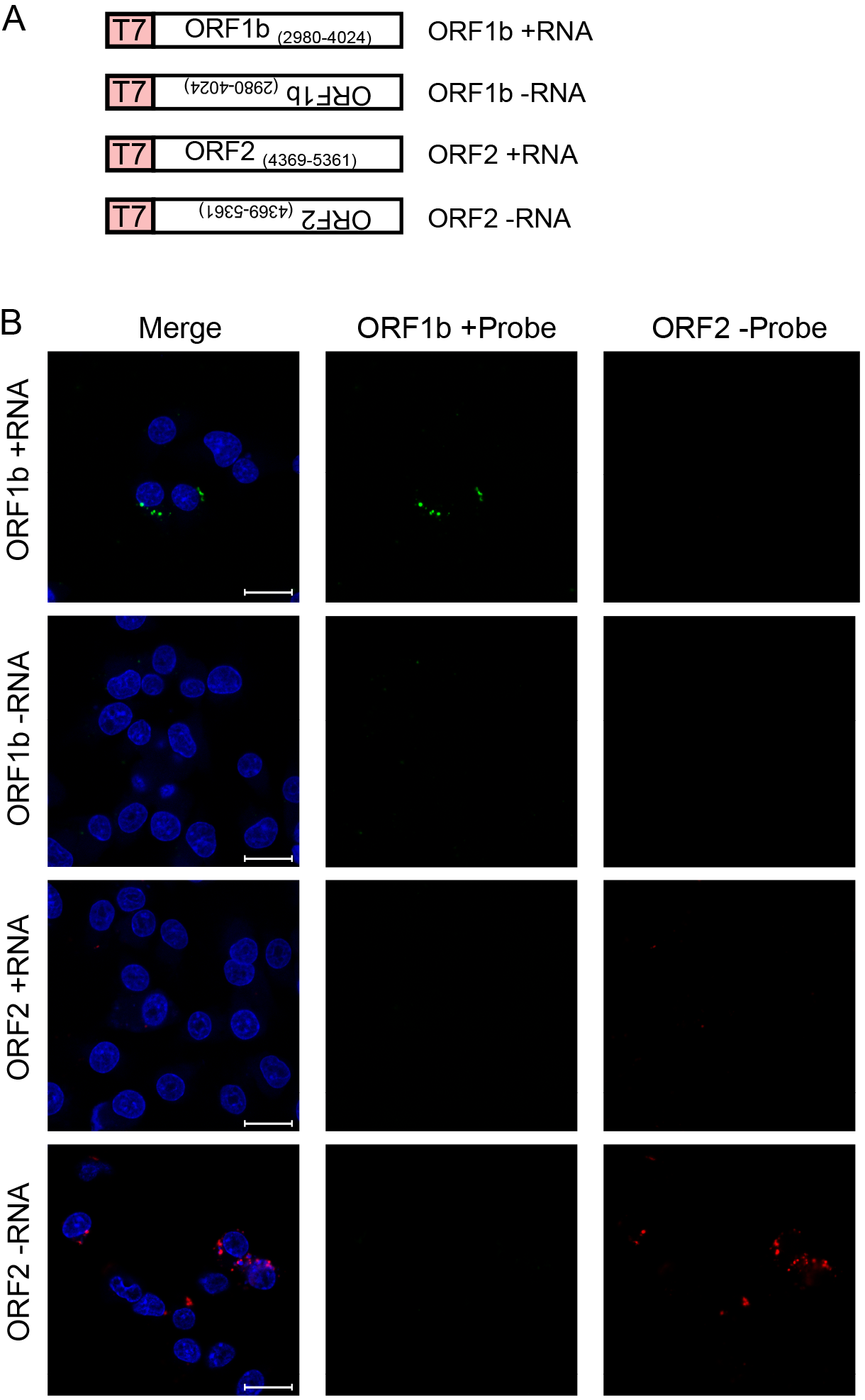

Supplement: Figure S3 — Strand specific FISH validation. [file jvi.00240-25-s0003.tif]

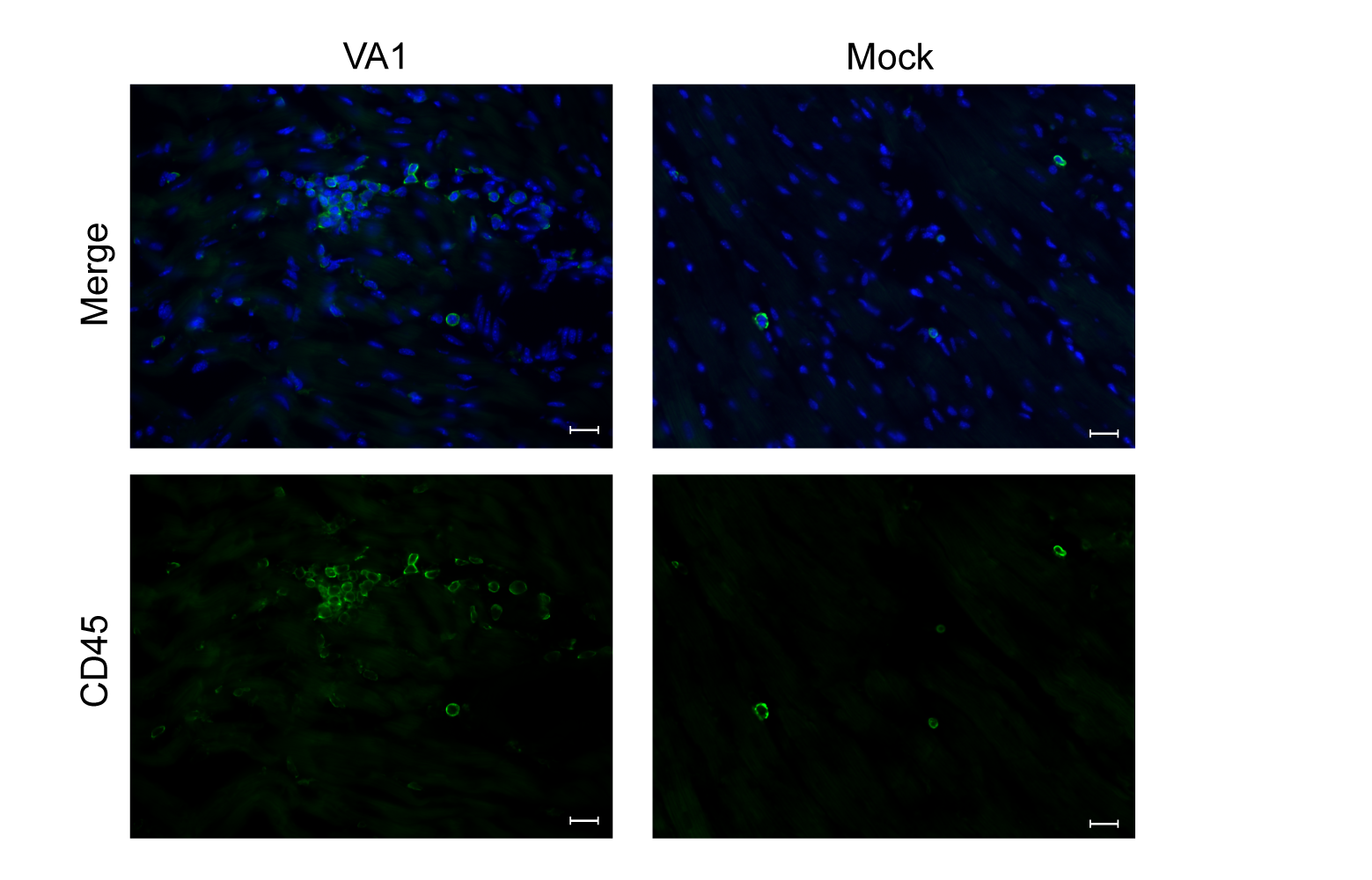

Supplement: Figure S4 — CD45 staining. [file jvi.00240-25-s0004.tif]

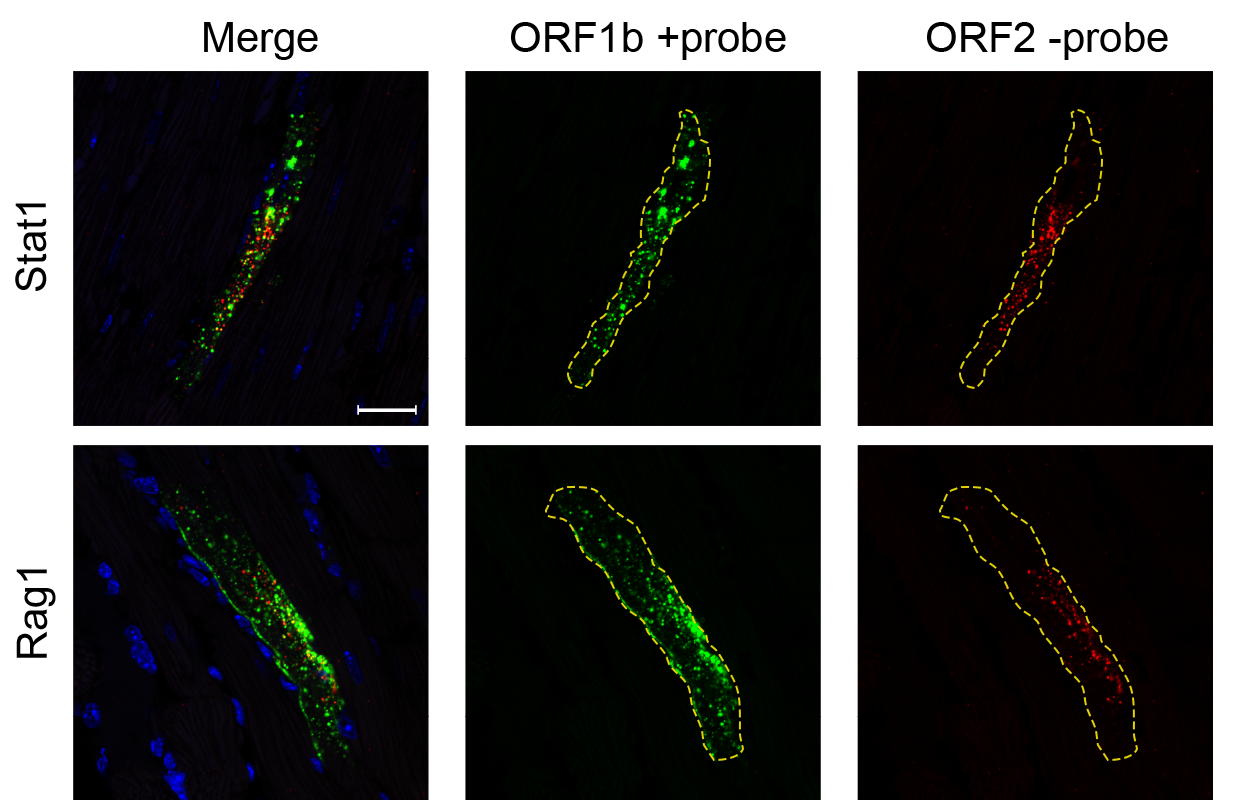

Supplement: Figure S5 — Negative stand RNA detection from Stat1 and Rag1 KO mice. [file jvi.00240-25-s0005.tif]
