# Supplementary material for: Novel murine model of human astrovirus infection reveals cardiovascular tropism
Source: J Virol. 2025 Apr 30;99(5):e00240-25. doi: 10.1128/jvi.00240-25 (PMC12090817; doi:10.1128/jvi.00240-25)
Supplement: Supplemental legends — Legends for Fig. S1 to S5. [file jvi.00240-25-s0006.docx]

**Supplemental Fig 1. VA1 is sporadically detected in low quantities in various tissues over a 21-day time course following inoculation**

Viral RNA copy numbers were measured by qRT-PCR from multiple additional tissues after IP inoculation of VA1 in wildtype A/J mice. Most tissues were undetectable at any time point, including the lung, kidney, ileum, mesenteric lymph node, and brainstem tissue. Dashed line represents the limit of detection.

**Supplemental Fig 2. Development of a** **fluorescent *in situ* hybridization (FISH) assay**

A fluorescent in situ hybridization (FISH) assay was developed using probes complementary to ORF2 of VA1. VA1-infected or mock-infected Caco-2 cells were stained with the VA1 probe and DAPI (blue), demonstrating specificity of the probes to virally infected cells. Scale bars represent 20 µm.

**Supplemental Fig 3. Development of a strand specific FISH assay**

(A) Controls for the strand-specific FISH was constructed using PCR. The T7 promotor was inserted 5’ to target regions, in either positive sense or negative sense orientation. These amplicons enable transcription of positive or negative sense RNA from each target region. (B) BHK-21 cells stably expressing T7 RNA polymerase were transfected with cDNA from each target region in either positive or negative senses. The ORF1b positive sense probe only produced signal when the BHK cells were transfected with the T7-cDNA fragment that yielded positive sense RNA to the target region. The ORF2 negative sense probe only detected negative sense RNA specific to the target region. Scale bars represent 20 µm.

**Supplemental Fig 4. Cellular infiltrates in heart tissue are CD45 positive**

Heart tissue sections from VA1 or mock-infected mice were stained for CD45. Foci of infiltrating cells were only identified in VA1 infected heart tissue and stained positive for CD45. Only sporadic detection of CD45 was detected in mock-infected heart tissue. 40x magnification, scale bars represent 20 µm.

**Supplemental Fig 5. Detection of negative strand RNA from heart tissue of Rag1 and Stat1 KO mice.**

Hearts from VA1 inoculated Stat1 or Rag1 KO mice were stained using strand-specific FISH probes. Both positive and negative sense strands were detected in heart tissue, demonstrating active replication of VA1. Scale bars represent 20 µm.
